# Development and performance of CUHAS-ROBUST application for pulmonary rifampicin-resistance tuberculosis screening in Indonesia

**DOI:** 10.1371/journal.pone.0249243

**Published:** 2021-03-25

**Authors:** Bumi Herman, Wandee Sirichokchatchawan, Sathirakorn Pongpanich, Chanin Nantasenamat

**Affiliations:** 1 College of Public Health Science, Chulalongkorn University, Bangkok, Thailand; 2 Faculty of Medical Technology, Mahidol University, Salaya, Nakhon Pathom, Thailand; The University of Georgia, UNITED STATES

## Abstract

**Background and objectives:**

Diagnosis of Pulmonary Rifampicin Resistant Tuberculosis (RR-TB) with the Drug-Susceptibility Test (DST) is costly and time-consuming. Furthermore, GeneXpert for rapid diagnosis is not widely available in Indonesia. This study aims to develop and evaluate the CUHAS-ROBUST model performance, an artificial-intelligence-based RR-TB screening tool.

**Methods:**

A cross-sectional study involved suspected all type of RR-TB patients with complete sputum Lowenstein Jensen DST (reference) and 19 clinical, laboratory, and radiology parameter results, retrieved from medical records in hospitals under the Faculty of Medicine, Hasanuddin University Indonesia, from January 2015-December 2019. The Artificial Neural Network (ANN) models were built along with other classifiers. The model was tested on participants recruited from January 2020-October 2020 and deployed into CUHAS-ROBUST (index test) application. Sensitivity, specificity, and accuracy were obtained for assessment.

**Results:**

A total of 487 participants (32 Multidrug-Resistant/MDR 57 RR-TB, 398 drug-sensitive) were recruited for model building and 157 participants (23 MDR and 21 RR) in prospective testing. The ANN full model yields the highest values of accuracy (88% (95% CI 85–91)), and sensitivity (84% (95% CI 76–89)) compare to other models that show sensitivity below 80% (Logistic Regression 32%, Decision Tree 44%, Random Forest 25%, Extreme Gradient Boost 25%). However, this ANN has lower specificity among other models (90% (95% CI 86–93)) where Logistic Regression demonstrates the highest (99% (95% CI 97–99)). This ANN model was selected for the CUHAS-ROBUST application, although still lower than the sensitivity of global GeneXpert results (87.5%).

**Conclusion:**

The ANN-CUHAS ROBUST outperforms other AI classifiers model in detecting all type of RR-TB, and by deploying into the application, the health staff can utilize the tool for screening purposes particularly at the primary care level where the GeneXpert examination is not available.

**Trial registration:**

NCT04208789.

## Introduction

Rifampicin Resistant Tuberculosis (RR-TB) is the single drug-resistant (DR) type where a mutation in the *rpoB* gene occurs. This becomes a focus on tuberculosis elimination, along with isoniazid and fluoroquinolone resistance [1]. Around half a million RR-TB cases around the world were diagnosed in 2018 [2]. Management of RR-TB is essential as it is linked to severe type of resistance, as reported that 78% of RR-TB belongs to MDR [2]. Pulmonary tuberculosis patients can spread the droplet easier than the extrapulmonary manifestation through a cough. Hence, a pulmonary RR-TB possesses a greater risk to be transmitted and rises a concern in public health.

Delayed diagnosis is associated with delayed treatment and severe clinical presentation but the diagnosis of drug-resistant tuberculosis is complex and prone to a procedural error. The existing rapid molecular test based on Nucleic Acid Amplification (NAAT) and Line Probe Assays reduce the waiting time, specifically in detecting RR-TB such as a study of Xpert performance in India [3]. This modality has been proven to save Daily Adjusted Life-Years (DALYs) particularly in TB-HIV patients [4]. But this is not without a problem. First, a low-resource setting is unable to perform this test as it needs particular requirements and maintenance including facility, devices [5], and human resources. Second, the pre-analytic procedure also affects the outcome, including the treatment of the sample/sputum, and a different source of specimens shows various results [6]. The phenotypic drug-susceptibility test (DST), considered the gold standard, is susceptible to errors such as incorrect inoculum preparation and different *in vitro* resistance criteria [7]. These high-cost and complex technologies, therefore, are one of the challenges in diagnosis.

The use of artificial intelligence (AI) has been acknowledged in the medical field including the use of machine learning and deep learning for clinical decision making and gain concern to alleviate the burden of disease screening. The scoring method to classify the disease is one of the approaches applied in the diagnosis algorithm. But the concern is, there are some variations of the criteria, and the diagnostic performance (sensitivity, and specificity) is lower than expected, such as TB Scoring in children [8]. The classifiers were then introduced including Logistic Regression, Support Vector Machine, Gradient Boost, Decision Tree, and Neural Network. The latter has gained interest as it has some advantages which outperform the other models [9]. The two most common Neural Network models are the Artificial Neural Network (ANN) model and Convolutional Neural Network (CNN) model. The multidimensional input such as image is commonly used in CNN whereas the ANN provides better performance in data or pattern recognition [10].

Clinical and demographic information plays a pivotal role in disease prediction and can be used for classification purposes, specifically for DR-TB classification. However, fewer studies are concerned about the use of these data as a predictor for decision-making due to scarcity and reliability. But nowadays, the data could be obtained from medical records and the extensive use of electronic records ensure sustainable data availability. Several previous models were developed based on clinical data using logistic regression, classification tree, or even ANN [11, 12]. However, several critical issues were found including a smaller data set and no validation with new participants. This study aims to develop a pulmonary RR-TB model classifier and deploy it to the application called CUHAS-ROBUST (Chulalongkorn-Hasanuddin Rifampicin Resistant Tuberculosis Screening Tool) using clinical and demographic parameters as the predictor. The authors assume that this application will possess a similar screening ability of any type of RR-TB (including the RR-TB occur with other Drug-Resistant TB such as MDR-TB) compared to existing rapid tests using the phenotypic DST as the reference standard.

## Methods

### Study design, data collection, and eligibility

This study is based on the diagnostic study using the cross-sectional approach. The data collection procedure was conducted in two steps. The initial step was intended for model building. The authors targeted medical records from the hospital managed under the Faculty of Medicine, Hasanuddin University, Indonesia. Patients who underwent phenotypic drug-susceptibility tests (DST) for tuberculosis (TB) from January 2015-December 2019 were recruited. This data was consecutively collected and sorted for eligibility from January-February 2020. All suspected drug-resistant tuberculosis (DR-TB) cases with the International Classification of Disease (ICD)-10. A.16 and A.15 refer to as pulmonary tuberculosis was extracted from electronic medical records. The age limit was at least 18 years old (as the people above this age can cooperate with the diagnostic procedure) with complete sputum DST results and parameters information. The authors excluded those patients who received prompt treatment which belongs to RR/MDR-TB drug regimen, confirmed by the information written on the TB form. This initial treatment is mainly given due to the long delay of DST results [13] and may mask the true DR-TB especially in low-resistance MDR [14]. For testing purposes, all participants with suspected DR-TB referred from the primary health care to the same hospital from January-October 2020 were tested with DST and model for CUHAST-ROBUST.

### Parameter definition

Based on associated factors of DR-TB particularly MDR [15] and other plausible mechanisms, the authors obtained information from the medical records of the eligible participants. This comprises of age at examination, gender, education level, universal health coverage, employment status, history of previous TB treatment, previous contact with positive DR-TB case, Brinkman Index for smoking assessment [16], history of drug abuse, alcohol consumption within the last one year, history of immunosuppressive therapy for more than 6 weeks [17], presence of Chronic Obstructive Pulmonary Disease (COPD), and the number of other chronic diseases besides diabetes mellitus (DM) and COPD. In clinical practice, these data were assessed in history taking and physical examinations by a physician, and other medical staff according to clinical pathways (such as American Diabetic Association for DM [18], or Global initiative of Lung Disease (GOLD) [19] for COPD. The authors collected other information including the body mass index (BMI), Human Immunodeficiency Virus (HIV) status, HbA1c, and sputum smear result before DST.

The HIV status is a mandatory test for any suspected drug-resistant cases. The rapid test of the anti-HIV antibody was conducted, followed by the Enzyme-Linked Immuno-Absorbent Assay (ELISA) for positive results [20]. The HbA1c is an examination that represents the average blood glucose within the last three months, conducted under the NGSP (National-Glycohemoglobin Standardization Program) standard [21]. As for sputum smear, this procedure was done using the Ziehl-Neelsen technique [22] and categorized based on the number of bacilli (negative, scanty, 1+ to 3+) [23]. These procedures were conducted in the Department of Clinical Pathology and Microbiology and confirmed by two or more staff to reduce inter-rater reliability issues. Laboratory technicians underwent the scheduled quality control programs every six months.

The authors obtained radiology data of Chest X-Ray and Computed Tomography (CT) scans including the number of cavitary, and extension of the lesion which were interpreted by two radiologists. The latter variable was defined as the segmentation of lung with pathognomonic lesion including cavitary, consolidation, nodules, fibrotic line, and atelectasis as these lesions are associated with DR-TB [24]. Lung was divided into three sections, yielding a total of six segments. One cavitary and nodule in the upper right lung is considered as one segment, but one cavitary in the upper right and left lungs considered as two segments. A detailed variable explanation is available in S2 Table.

### Reference standard

The phenotypic drug-susceptibility tests (DST) result was preferred over the GeneXpert as this is the standard diagnosis for Rifampicin-Resistant Tuberculosis (RR-TB) despite it is a time-consuming procedure [25]. The GeneXpert relies on the detection of the *rpoB* gene which is associated with RR-TB but has a sensitivity and specificity of 87.5% and 100% according to a study comparing a pulmonary sample of phenotypic versus DST in India [26]. The Lowenstein Jensen (LJ) has been implemented as the standardized method and less susceptible to cross-contamination, compare to the liquid method [27]. A proportion method is a protocol implemented over the last five years in these centers. Compared to the critical concentration method, the latter method is prone to underdiagnosis due to different responses of minimum inhibitory concentration in comparison with the standard strain [28]. A standard procedure for treating the sputum and DST process was applied. Two samples of the morning and random sputum were digested with 1% N-acetyl-L-cysteine and 2% sodium hydroxide. The N-acetyl-L-cysteine reduces the viscosity of mucoprotein solutions in-vitro, and the sodium hydroxide reduces any contamination of culture [29]. All samples were mixed with the solution, vortexed, and incubated for 15 minutes. Centrifugation was conducted and the deposit ready to be inoculated. A 0.2 ml of suspension was embedded in the LJ medium. The initial LJ culture is conducted to identify whether there is a presence of Mycobacterium tuberculosis among all samples collected. The technician observes any growth, daily within the first week, and once per week until 8 weeks. The absence of growth indicates there is an error in the procedure (from sample collection to culture) or the patient is not infected with Mycobacterium tuberculosis. The culture with positive growth was then tested for DST with rifampicin concentration 1μg/mL [30]. The cutoff of this method for resistance is >1% [31].

### Model development for index test

The parameters were selected and assessed for completeness. Participants with insufficient information were excluded. The full model with 19 parameters (consists of a group of age, gender, education level, health insurance coverage, current employment status, history of drug abuse, contact with DR-TB, HbA1c level (with cutoff 6.5), history of previous TB treatment, HIV status, Brinkman Index, alcohol drinking, prolonged immunosuppressants use, number of chronic diseases, BMI level, COPD, sputum smear level, number of cavitation and extension of the lesion) and models with eight parameters were created (short model). The short model omits parameters with higher recall bias, leaving only age, gender, BMI, HbA1c, number of cavitary, sputum smear level, and extension of the lesion in the lung segment as the predictors. A discretization of the variables was performed to boost the model performance [32] and to be relevant with the associated factors including a group of age (cutoff 40 years old), BMI, and HbA1c (cutoff 6.5). Another model was developed using variables that had significant bivariate associations (S1 Table).

The model building was aimed to create a binary classifier for RR-TB. Any other DR-TB that includes rifampicin-resistant, such as Multidrug (MDR)-Resistant TB were also defined as positive results. Other than these cases were treated as negative, including non-rifampicin-resistant.

The Artificial Neural Network (ANN) is commonly used in medical classification and is based on how the neuron cells transmit the information. This model transfers the input to hidden layers with certain weights and activation functions. and the model can adjust for error through propagation [33]. The ANN models were constructed using the R program [34], a summary of model construction, and mathematical models are written as S3 Table). Normalization was conducted using the min-max function. To assess the appropriate training size for ANN, the authors evaluated the training size and ROC (Receiver-Operating Characteristic) value, and the prior train-test splitting was appropriate to converge the ROC value (S1 Fig). A seed was set followed by splitting the data from the initial stage for training and testing splitting with 85%:15% based on the convergence of ROC values according to training size. The “neuralnet” package was utilized to build the models, which are based on resilient backpropagation [35]. Two hidden layers were determined for all models with maximum steps 10^5^ for the full model and 10^6^ for the short model and model with significant bivariate analysis. The activation function of logistic/sigmoid was applied to all layers. Repetition was done 20 times for the full model and 10 times for the short model with the default threshold of 0.01. Logistic loss/Log loss was calculated in the “MLmetrics” package and the best models were shown with the plots [36].

A total of six ANN models were created and saved as.rds files. The author set a cutoff of 0.5 probability for the result interpretation. The name and detailed structure of the ANN models are available in S5 Table. These ANN models were tested with 15% data from the main data set. The Area Under Curve (AUC), Accuracy, Sensitivity, Specificity, and Log loss of the six models according to the testing data performance were summarized and available in S7 Table.

The authors also constructed four different groups of classifiers, the Decision Tree (DT), Random Forest (RF), Logistic Regression (LR), and Extreme Gradient Boost (XGB). The 85%:15% data splitting was conducted. The best model for LR and DT model was determined from the sensitivity value of testing data. For RF and XGB model, the lowest error of the five-fold cross-validation was used for selecting the best model. A total of additional 12 models were constructed. (detailed information on S6 Table).

### Screening procedure

The second stage of data collection involves consecutive participants recruitment from the same centers. All suspected DR-TB cases from outpatient and inpatient units were recruited from January 2020 until October 2020 following similar eligibility criteria. The DST was conducted independently by the laboratory technician without prior knowledge of model results. Cases with no growth from multiple samples in the initial LJ culture (until 8 weeks) will be excluded as it indicates an error in laboratory procedure or the sample collection, or the patient was not infected with TB. The performers of the model tested the eligible patients in two categories. First, the participants who underwent DST and the DST result have not been issued. Second, those participants that scheduled to DST. Eligible participants that were scheduled for DST but died were excluded. This procedure ensures that both assessors were blinded to each test result. The time interval between the model test and DST was 1 day –6 months.

For final validation using the prospectively collected data, the authors developed the framework of the CUHAS-ROBUST application which is ready to be integrated with the core model. There were two options to test the performance of the models with the prospective data. First, the individual testing, where each model is embedded into the application and the parameters are inputted manually. Since the authors built 18 models, batch testing with all models was preferred where a prediction of whole data could be performed in a single step. S9 Table show the accuracy, sensitivity, and specificity of 18 models, tested by prospectively collected data. From 18 models, the model with the best sensitivity, specificity, and accuracy exceeds 80% was deployed into the CUHAS-ROBUST application using the “Shinyapps” platform in R-Studio for further use in other settings. This web-based application provides a prediction tool with additional features for parameter calculation including BMI calculator, Brinkmann Index, and HbA1c estimation.

### Analysis and sample size

The resampling bootstrap method was executed to overcome the small number of prospective participants [37] particularly amid COVID 19 pandemic. The bootstrap method ensures that the new data sets represent a similar confidence interval to the prospective data using the resample program [38]. Sensitivity is the main focus as the function of a screening test is to detect as many true-positive cases as possible. The estimated prevalence of RR-TB among suspected RR-TB is 10% according to a study in West Java, Indonesia [39]. Since the recruitment of prospective study may not follow the real prevalence as the hospital-based study may recruit more sick participants, therefore the authors set a hypothesis which not depends on the prevalence. The null hypothesis of 85% sensitivity (P0) and 90% as an alternative hypothesis (P1) (approximate power 90% (1-β) and type I error/α as 5%) would yield a total of 471 participants [40]. The hypothesis can be written as H0: Se = P0 versus H1: Se ≠ P0 (or Se = P1) with the formulation as follows:

$$n=\frac{{\left[Z\frac{\alpha }{2}\sqrt{P0(1-P0}+Z\beta \sqrt{P1(1-P1)}\right]}^{2}}{{\left(P1-P0\right)}^{2}}$$ (1)

The authors assessed the completeness of data and conducted an initial descriptive and bivariate analysis for parameter selection. As the outcome is binary (yes or no), therefore, no specific effort to handle indeterminate results. As completeness of the data was one of the eligibility criteria, the missing data imputation would not occur.

### Ethical statement

This study has been registered for clinical trial number NCT04208789 with full protocol available on the clinicaltrials.gov website. The approval from the Institutional Review Board was granted from the Faculty of Medicine Hasanuddin University (expedited) and The Research Ethics Review Committee for Research Involving Human Research Participants, Chulalongkorn University (exempted).

The researchers were granted access to both electronic and conventional medical records, indirectly, which began in January 2020. Data was collected based on a request in a limited number per day to prevent the disruption in health service while the data was being accessed. The hospital staff retrieved the desired information from the electronic system without identifiable information such as name, and address, and other detailed information, and handed it to the researcher. If the electronic system provides insufficient information, the hospital staff (not the researcher) will open the hardcopy version of medical records and obtain the information manually.

## Results

**Fig 1:**
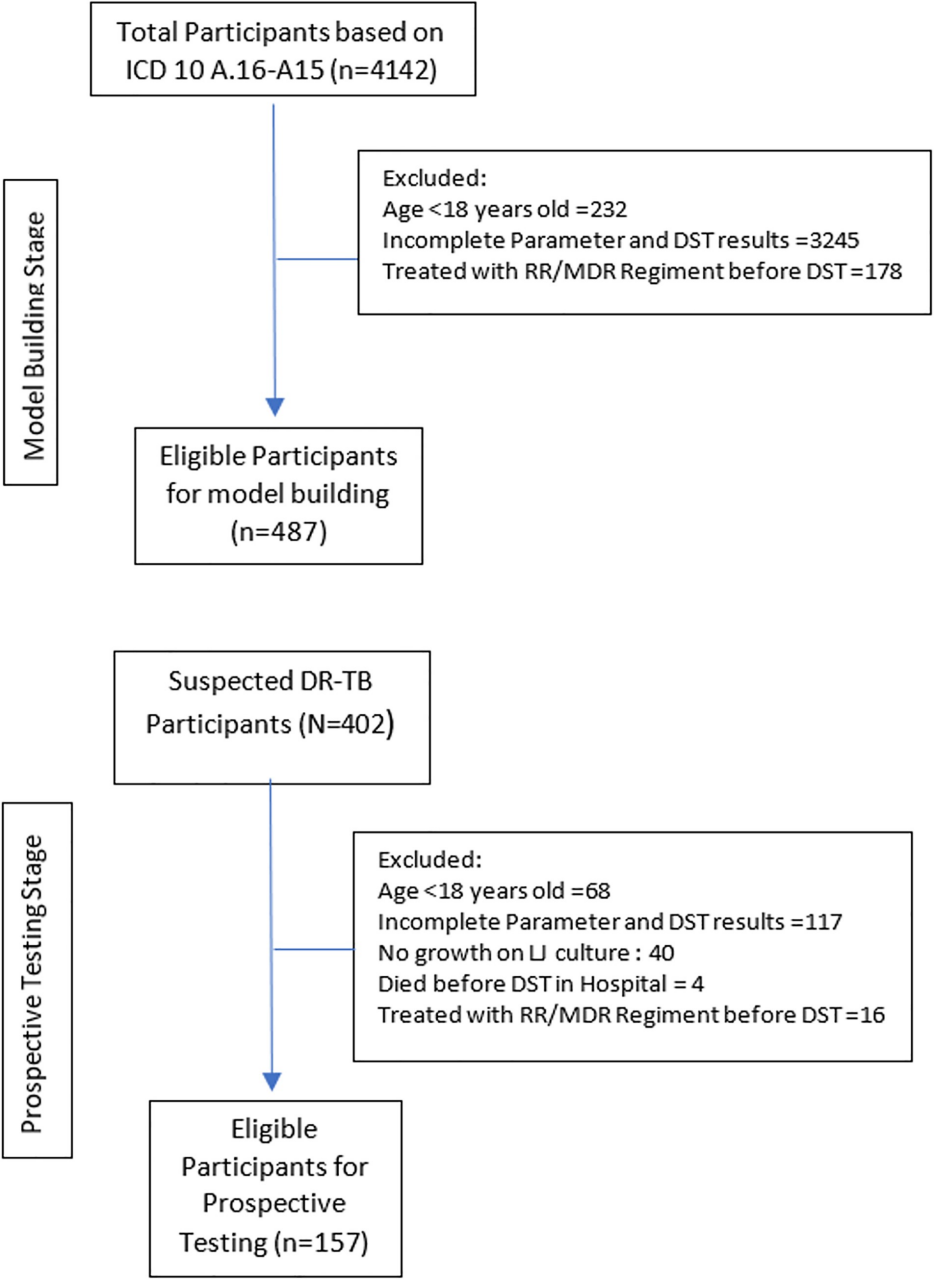
Flowchart of participants recruitment. The figure consists of two diagrams. The upper diagram illustrates the selection of data for model building and the lower diagram for the prospective data collection. Notice that some prospective participants were excluded due to procedural reasons. As 40 people were excluded for showing no growth on Lowenstein Jensen culture after eight weeks. Four in-patient participants were scheduled to have a DST but later pronounced death before DST could take place.

After eligibility screening, a total of 487 data involved in data building consists of 89 participants of DR-TB with rifampicin-resistant (32 participants were MDR-TB) and 398 participants showed no drug-resistant (Table 1). A total of three non-rifampicin-resistant (isoniazid-resistant) cases were observed but excluded due to data incompleteness. Most of the treatment given for the participants was a combination of Streptomycin + Levofloxacin + Ethambutol, after the DST procedure. Detail of participant recruitment is illustrated in Fig 1.

**Table 1:** Participant’s characteristic in model building data (n = 487).

Variable	Subset	RR + MDR (n = 89)	Non-RR (n = 398)	p value
Gender	Male	41	132	0.021
	Female	48	266	
Age (year)	<40	30	210	0.001
	40 and above	59	188	
	Mean ± SD	44.06 ± 11.57	39.59 ± 13.84	
Education	Illiterate	4	13	0.662^&^
	Primary Education	19	95	
	Secondary Education	64	271	
	College degree and above	2	19	
Universal Health Coverage	Covered	70	331	0.313
	Uncovered	19	67	
Current Employment Status	Employed	34	168	0.488
	unemployed	55	230	
History of Drug Abuse	Never	87	396	0.154^&^
	Yes	2	2	
Contact with positive DR-TB case	Never	61	381	<0.001
	Yes	28	17	
DM status	No	40	252	0.001
	Yes	49	146	0.004#
	Mean ± SD of HbA1c	7.33 ± 1.86	6.91 ± 1.95	
History of Previous TB treatment	Never	27	299	<0.001
	Yes	62	99	
HIV status	Reactive	26	77	0.039
	Non-Reactive	63	321	
Brinkmann Index	Never Smoke	53	340	<0.001
	1–600	27	55	
	>600	9	3	
Drink alcohol within one year	Never	86	395	0.078^&^
	Yes	3	3	
Immunosuppressants use > 6 weeks	Never	85	390	0.245^&^
	Yes	4	8	
Number of Chronic Disease	Median ± IQR	0 ± 0	0 ± 0	<0.001^
	Min-Max	0–2	0–2	
Body Mass Index (kg/m2)	<18.5	49	74	<0.001
	18-5-<23	28	194	
	23–25	6	71	
	>25	6	59	
Adherence to Previous TB treatment	Yes	30	84	<0.001
	No	32	15	
Diagnosed as COPD	Yes	21	40	<0.001
	No	68	358	
Sputum Smear level	Negative or Scanty	3	285	
	1+	37	99	
	2+	32	9	<0.001
	3+	17	5	
Presence of Cavitation	Yes	55	77	
	No	34	321	<0.001
	Median Number ± IQR	0 ± 2	0 ± 0	<0.001^$^
	Min-Max of Cavitation	0–4	0–4	
Extension of Lesion	Median ± IQR	3 ± 1	2 ± 2	<0.001^
	Min-Max	1–4	0–4	

Abbreviation: COPD (Chronic Obstructive Pulmonary Disease), DM (Diabetes Mellitus), DR TB (Drug-Resistant Tuberculosis). DST (Drug Susceptibility Test), HbA1c (Hemoglobin Glycated 1c) HIV (Human Immunodeficiency Virus), IQR (Interquartile Range), Max (Maximum), MDR (multidrug-resistant) Min (Minimum), SD (Standard Deviation). All tested with Chi-Square, except (& = Fisher Exact).

# is a Mann-Whitney U test for the difference between HbA1c values.

$ is a Mann Whitney test for the difference of cavitation number between the group

^ tested with Mann Whitney. The baseline for prospective testing data provides as S1 Table.

As for the prospective data collection, a total of 157 participants suspected DR-TB (23 with MDR-TB and 21 RRTB) were recruited. All negative results were sensitive/no drug-resistant. The turnaround time for culture growth ranging from 2–8 weeks, and 7–11 weeks for a drug-susceptibility test. Similar combination therapy was given to all positive cases.

**Fig 2:**
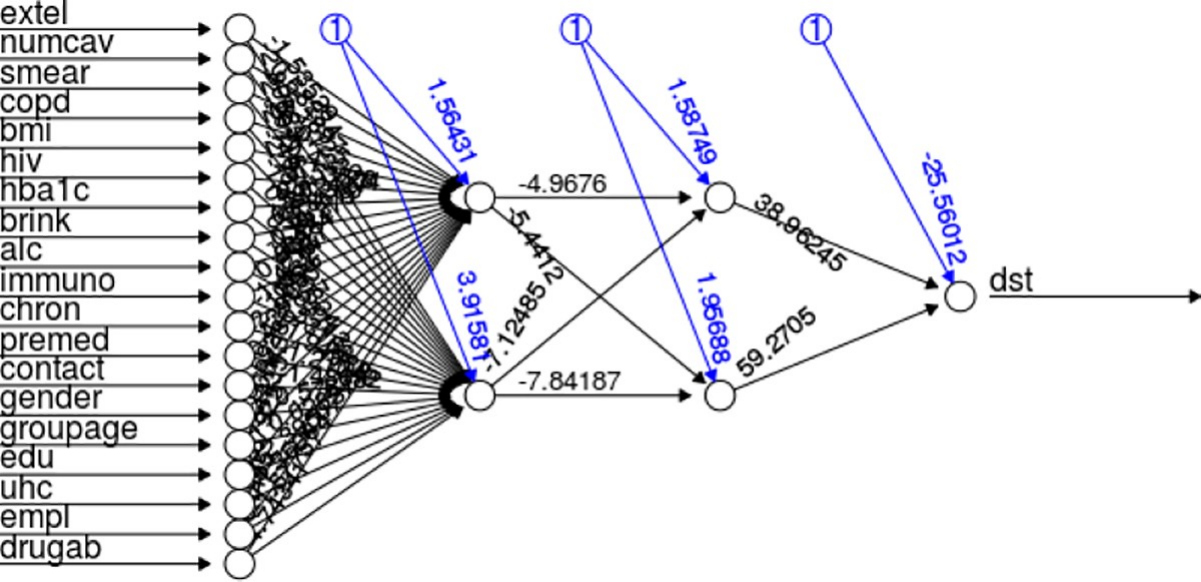
ANN structure of the CUHAS-ROBUST model. This figure depicts the ANN model with 19 parameters, two hidden layers with two nodes in each layer and the blue lines show the weight of bias in each node.

The authors built six models of ANN with a summary of the performance according to 15% of the data for model building presented in Table 2. The ANN Short model with two hidden layers and two nodes in each layer (ANN Short 2–2) outperformed 6 other ANN models in terms of accuracy, sensitivity, and specificity (96%, 84%, and 100% respectively), followed by ANN full model with the similar structure (Fig 2).

**Table 2:** Performance of Artificial Neural Network model with 15% training data (N = 73).

Model	TN	TP	FP	FN	%Acc (95% CI)	% Sens (95% CI)	% Spec (95% CI)	LogLoss	AUC
2.2 Full	51	16	3	3	92 (83–97)	84 (60–97)	94 (85–99)	1.53	0.96
2.1 Full	51	13	3	6	88 (78–94)	68 (43–87)	94 (85–99)	1.56	0.94
2.1 Short	52	13	2	6	89 (79–95)	68 (43–87)	96 (87–99)	0.91	0.95
2.2 Short	54	16	0	3	96 (88–99)	84 (60–97)	100 (93–100)	0.18	0.99
Bivariate 2–2	50	16	4	3	90(81–96)	84 (60–97)	93(82–98)	2.81	0.96
Bivariate 2–1	51	16	3	3	92(83–97)	84 (60–97)	94 (85–99)	1.25	0.98

Abbreviation: Acc = Accuracy; AUC = Area Under Curve; CI = Confidence Interval; FN = False Negative; FP = False Positive; TN = True Negative; TP = True Positive

A further validation using prospective and bootstrap data were conducted. In prospective data, other models show very high specificity, particularly logistic regression in prospective data (99%) but lower sensitivity (<50%). Interestingly, the ANN short model did not have a good specificity. Overall, the ANN full 2–2 model is superior with 90% accuracy, 84% sensitivity, and 92% specificity (S9 Table) and outperformed the other 17 models. Despite having lower specificity compared to the Logistic Regression Full model, ANN full 2–2 model maintain its highest performance with 88% accuracy, 84% sensitivity, and 90% specificity on bootstrap data, shown in Table 3.

**Table 3:** Performance of all models on bootstrap data.

Model	True Negative	False Positive	False Negative	True Positive	%Accuracy (95% CI)	% Sensitivity (95% CI)	% Specificity (95% CI)
DT Full	312	24	75	60	79(75–83)	44(36–53)	93(90–96)
RF Full	314	22	101	34	74(70–78)	25(18–33)	93(90–96)
LR Full	332	4	92	43	80(76–83)	32(24–40)	**99(97–99)**
XGB Bivariate	316	20	101	34	74(70–78)	25(18–33)	94(91–96)
ANN 2–2 Full	301	35	22	113	**88(85–91)**	**84(76–89)**	90(86–93)

Abbreviation: ANN (artificial Neural Network), DT (Decision Tree), GB (Gradient Boost), LR (Logistic Regression), RF (Random Forest), XGB (Extreme Gradient Boost)

## Discussion

The Artificial Neural Network (ANN) full model outperforms other models in terms of accuracy, and sensitivity. The ANN is known for its benefit in classifying disease. A review shows that the neural network can be built as a single model or assembled with a different classifier (such as merging ANN with Regression Tree to be one whole model) and demonstrate diverse results [41]. This project focuses on ANN as a single classifier of any type of RR-TB, and not expanding its assessment for specific MDR-TB only and other types of DR-TB.

Comparison to other studies with a similar design was done but some studies mainly focused on MDR-TB. A study to build an MDR-TB classifier was conducted using Logistic Regression of fewer clinical data including the history of the previous TB, contact with MDR-TB, presence of cavitary in X-Ray and abnormal physical examination. The performance varied across the different cut-offs where total score two as the cut-off value demonstrates 85.6% accuracy, 60.8% sensitivity, and 87.5% specificity [11]. Another model with ANN involved clinical data was built with similar steps except for the hyperbolic tangent activation function. The clinical information used for the parameter including age, gender, marital status, history of imprisonment, previous TB treatment, contact, smoking, drinking alcohol, and cavitary in radiology. The sensitivity to distinguish the drug-resistant from the non-drug resistant reached 95.7%, 86% specificity, and 88.1% accuracy but the deteriorating performance was observed when tested for MDR prediction 82.8% sensitivity, 91.2% specificity, and 85.3% accuracy. This study developed the Classification and Regression Tree (CART) of MDR where the performance in all three indicators was even below 60% (59% Sensitivity, 39.3% Specificity, and 50.5% Accuracy. These models obtained 280 data for model building and tested with cross-validation of the data used for model building, something that enhances the superiority of the current study [12].

The strong aspects of this study are the data collection method and the reference test. The authors perform rigorous methods of screening the eligible participants through electronic medical records with ICD code and elaborates standard procedures and quality control applied. The electronic medical record system boosts the screening process and preserved data quality [42]. The exclusion was made to the one who received the prompt treatment before DST. Despite different DST procedures were introduced at a certain time, the authors selected the participants who underwent the DST with the LJ method, reducing the heterogeneity of DST results due to different processes. In this study, all the negative results showed a sensitive result to all drug regimens.

Stricter eligibility criteria reduced the possible participants to be recruited in the study. A rule of thumb to calculate the sample size of the ANN is at least 10 samples per number of weights in the ANN structure despite one study suggests 50 samples [43]. By using the function to calculate the number of weights in ANN, the model with 8 parameters and two hidden layers with each of the layers consists of two hidden nodes needs a minimum of 270 data. The ideal number is 1350 data and this number is inflated when more parameters are introduced to the structure. Very low samples affect the prediction performance especially the log-likelihood-based measure in the learning curve. The authors notice that this issue is inevitable particularly in medicine where it is less likely to attain a bigger sample, except when conducted in homogenous multiple centers.

The authors emphasize the point that data processing plays a pivotal role. The discretization technique and parameter selection are essential. ANN short model with fewer variables that prone to bias such as variables from history taking provides better specificity and accuracy despite inconsistent findings shown in prospective and bootstrap data. Some variables were identified to be irrelevant despite several reviews show association. Alcohol drinking is forbidden in predominantly Muslim countries like Indonesia and this variable is prone to bias as people will reluctant to admit it [44], thus explaining the inconsistent association in training data and prospective data. Rather than providing a binary response, Brinkmann Index is the quantitative way to assess the smoking behavior and accommodate any smoking cessation impact from the past despite its dubious reliability. Most of the chronic diseases observed in the participants are hypertension, kidney disease, and heart disease. Breast cancer was observed in one patient. Patients with multiple complications or remarkably sick are often seen in participants on hospital-based recruitment [45], therefore, implying a full model may overestimate the prediction in a patient treated at the first-line healthcare system which tends to have less complication. The use of immunosuppressant agents identified in the study is the use of steroids, either oral or inhaled. Only one participant was given anticancer medication in prospective data. The authors consider body mass index, as it shows an inverse correlation to the rifampicin concentration in the body [46]. Another point is the authors did not focus on the DM status but the latest Hba1c point [47]. This is important as some of the DM patients may have controlled HbA1c which possibly reduces the occurrence of DR-TB. Furthermore, the authors provide the HbA1c estimation, but these estimate values were not considered for prediction. The authors provide this estimation in the application to expand the generalizability of CUHAS-ROBUST in the health service that unable to perform the NGSP-certified HbA1c. Regarding the radiology and sputum smear results, the authors treated the radiological finding as categorical input, not a high-dimensional input. Several studies were conducted using the radiological finding as a high-dimensional input (image) and predicted with complex neural network models such as Convolutional Neural Network. Nevertheless, it showed a lower performance [48–50]. Some pre-processing techniques and device settings affect the element of radiology image [51] including intensity, shape, and texture of the lesion portrayed in radiology film, despite digital image processing has been endorsed to tackle this issue. But there was one point where a COVID 19 patient was predicted resistant due to extensive consolidation on CT-scan results and this variable is highly important in some models. The authors suggest selecting radiology features that represent active tuberculosis including cavity, consolidation, or parenchymal infiltrate rather than including all features. This study is not considering other significant parameters including inflammatory markers, despite it has the possible predictive ability (such as C-Reactive Protein) as it is not routinely performed [52].

The authors elaborate on the model building process and applied a similar data splitting procedure when constructing another model. This study implemented normalization using min-max techniques [53] and it accelerates the model learning by simplifying the input to a certain range [54]. Identifying the number of training sizes and using multiple diagnostic parameters were performed to select the best model. However, in ANN model learning, the performance of the model was evaluated using the holdout technique, treating the testing data as the validation procedure compares to other models using cross-validation results and error rate. Using holdout validation excludes some of the data that perhaps suitable for training, therefore this method’s performance relies on the choice of data splitting, and repeated testing is suggested to take the average performance [55].

Backpropagation is the way the neural network learns by tuning the error of the weight neural network connection. There are some algorithms of backpropagations, including Levenberg-Marquardt, conjugate gradient, and resilient back-propagation. This study applies the resilient backpropagation in the neuralnet default package as it provides better performance in testing data (particularly accuracy), while other techniques may provide faster learning [56].

This study is still relying on the solid culture method rather than the liquid culture method which was recently applied in clinical practice. However, liquid culture is prone to contamination. Combining different DST confirmation introduces heterogeneity of the results, something which this study avoid.

A sampling bias still likely occurred in this setting. There is a question of whether the dataset truly represents the total population. Representativeness is one of the crucial points and the bigger number of samples may be linked to better representativeness. But the author acknowledged that there is no single dataset that perfectly represents the true population. The dataset is similar in terms of gender proportion in comparison to the total population, although the authors were unable to test the representativeness of other variables between the dataset and total data as most of the variables in the total population were incomplete (such as representativeness in a specific group of disease, ethnicity, or domicile, or other stratification factors). In machine learning model development, there is an essential issue of whether the prevalence of interest outcome in the dataset should be similar to the true population or not. The imbalanced data is where the proportion of the case is not balanced, either lack of positive cases or negative cases, and it affects the model performance [57]. By assessing the prevalence of disease between the dataset and total data, the author noticed a difference in the RR-TB prevalence of the dataset and total data (18.27% vs 10.26%). Indeed, there is an imbalance of data in this situation but the prevalence of the dataset shows a better proportion and closer to 50:50 distribution, which then the non-representativeness due to the different prevalence is permissible. There are several techniques to deal with the imbalance of data and one of the common approaches is Synthetic Minority Oversampling Technique or SMOTE. The SMOTE will add synthetic data to the minority group (in this situation, the number of the positive case), or omitting the sample of the majority group which is the negative case [58]. As the prevalence is low, it is unlikely to synthesize more positive cases and the possible option is to listwise more negative cases to achieve a nearly balanced proportion. But this would induce another threat as the number of the dataset will be smaller. To sum up, the author acknowledged that the representativeness of the dataset with the total population is questionable and the initial tests was insufficient to prove the representativeness. But the difference of prevalence between the dataset and the total population is acceptable in machine learning, particularly when the dataset is closer to balanced data and it can boost the model performance.

Validation with new data is the point that the authors intended to address because the machine learning model tends to memorize the model and overfitting may occur [55], but technical issues hinder the procedure. The COVID 19 pandemic changes the current practice where the referral process is affected the most, leaving only people with the highest possibility of DR-TB can be referred for the GeneXpert and DST testing. This was worsened by the allocation of GeneXpert for COVID 19 diagnosis. Moreover, rigid inclusion criteria (completeness information) contribute to a lower number of eligible prospective participants. This is the underlying reason why the prevalence of RR-TB in this study is higher compare to the study in Indonesia [39]. Hence, the bootstrap method was the only way to yield a sufficient number for testing. Compare to GeneXpert’s result from a multicenter study based on DST, the ANN model deployed to CUHAS-ROBUST shows lower sensitivity (84% vs 87.5%), but GeneXpert results vary across the personal history of tuberculosis and smear level [59].

Caution should be taken when using this application in different settings as this model was developed using single province data and tested with the prospective data from the same center. This is a true limitation of this study and hence, the nationwide trial of CUHAS-ROBUST should be conducted to assess the true performance. The main reason why this study was conducted in several centers in one province was a trade-off between the heterogeneity (medical procedures, locality contexts, and human resources to collect the parameter needed) and generalizability. The authors acknowledge that there would be disparities in healthcare service, particularly the quality of healthcare modalities and medical staff’s reliability which affected the quality of data. One variable that is affected by different healthcare quality is diabetes mellitus (DM). The diabetes prevalence in Jakarta is the highest in Indonesia compared to the study center. With better accessibility of DM treatment in the capital city, DM patients in Jakarta may have better glycemic control. A study shows that an individual who lives in the capital and having multiple comorbidities, may afford or get access to medication, which then reduces the likelihood of getting the disease compared to the individual with the same comorbid who live in a remote area [60]. Hence it is unlikely to see a strong significant association between DM and drug-resistance compared to the results in the study center. Furthermore, the authors might assume that overestimation may exist from the model because of this reason. Another point is the discrepancy of quality of laboratory and radiology examinations which introduces heterogeneity and it’s a challenge to standardize the procedure as the authors relied on the retrospective data. The single-center system may reduce the possible heterogeneity arise from these disparities. But a multicenter study may introduce higher data variability which essential in model building. The locality context exists when several associated factors may not be relevant in certain regions, particularly sociodemographic factors related to religion, values, and norms of the dominant ethnic group. One context related to the local norm is alcohol consumption. The study area is predominantly Muslim, where the distribution and consumption of alcohol are prohibited and/or restricted, hence this factor was omitted as a possible predictor. Different considerations could be taken when this study is conducted in the predominantly non-muslim area. A larger study area with various local factors enforces more parameters to be included, which affects the screening procedure as many parameters should be obtained. If accommodating too many factors as the parameter, future screening will be inconvenient. It is really common to observe the deteriorating diagnostic performance for diagnostic tools after implemented to the larger population. It is either due to the aforementioned factors or the difference in the actual prevalence of the disease that was used to justify the initial sample for screening performance. Furthermore, different prevalence might affect the implicit threshold of a physician to determine and interpret the diagnostic results [61]. This implicit threshold also arises from the prior knowledge of rapid test results as people who underwent the DST must undergo the GeneXpert test and positive cases gain more attention compared to those negative cases. A study in machine learning, therefore, should consider all of these factors and be conducted with a rigid operational definition, reliable measurement tools and procedures.

Deployment of the model to an application, the CUHAS-ROBUST generates a possibility that the RR-TB screening can be done in a primary-care setting where the suspected DR-TB case comes for screening. By implementing this screening in primary care, two objectives could be achieved. The first is evaluating the screening ability of the patient (which commonly appears healthier than the patient at a higher healthcare facility) at the first screening point, second is to enhance a faster prompt treatment and surveillance (including tracing and screening). An open-system for CUHAS-ROBUST should be considered to facilitate the user’s contribution, including providing new data for the model update which can improve future performance. The provision of supporting modalities (including X-ray) in primary-care is another recommendation to maximize the CUHAS-ROBUST screening.

Despite the CUHAS-ROBUST application with the ANN model provides a lower sensitivity in comparison with the Xpert test for pulmonary RR-TB screening, the authors successfully describe crucial issues for further improvement, particularly in data collection and pre-processing.

## Supporting information

S1 Fig Training size according to ROC value showing a convergence of training and testing data at more than 400 training data. (DOCX)

S2 Fig Participants flowchart. (DOCX)

S3 Fig The Artificial Neural Network structure of full model with two hidden layers and two nodes in each layer. The blue lines indicate the bias of each node.
(DOCX)

S4 Fig Sensitivity specificity of selected model (Artificial Neural Network Full Model 2–2). (DOCX)

S5 Fig Receiver operating characteristic curve of selected model (Artificial Neural Network Full Model 2–2). (DOCX)

S6 Fig Precision recall of selected model (Artificial Neural Network Full Model 2–2). (DOCX)

S7 Fig CUHAS-ROBUST interface. (DOCX)

S1 Table Participant’s characteristic in model building data (n = 487). (DOCX)

S2 Table Descriptive statistic of prospective data (n = 157). (DOCX)

S3 Table List of variables. (DOCX)

S4 Table Mathematical equation of the model. (DOCX)

S5 Table Structure of the Artificial Neural Network Model (ANN) (R script available upon request). (DOCX)

S6 Table Structure of other classifiers (R script available upon request). (DOCX)

S7 Table Performance of Artificial Neural Network model with 15% training data (N = 73). (DOCX)

S8 Table Performance of other models with 15% training data (N = 73). (DOCX)

S9 Table Performance of all models from prospective data (N = 157). (DOCX)

S1 File (ZIP)

S2 File (ZIP)

S1 Dataset (ZIP)
