# Supplementary material for: Development and performance of CUHAS-ROBUST application for pulmonary rifampicin-resistance tuberculosis screening in Indonesia
Source: PLoS One. 2021 Mar 25;16(3):e0249243. doi: 10.1371/journal.pone.0249243 (PMC7993842; doi:10.1371/journal.pone.0249243)

S1 Fig. Training size according to ROC value showing a convergence of training and testing data at more than 400 training data.


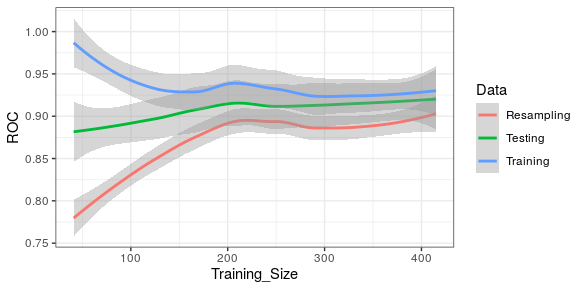

Supplement: S1 Fig — (DOCX) [file pone.0249243.s001.docx]
