# Supplementary figures and images for: Development and performance of CUHAS-ROBUST application for pulmonary rifampicin-resistance tuberculosis screening in Indonesia
Source: PLoS One. 2021 Mar 25;16(3):e0249243. doi: 10.1371/journal.pone.0249243 (PMC7993842; doi:10.1371/journal.pone.0249243)

S2 Fig. Participants Flowchart.


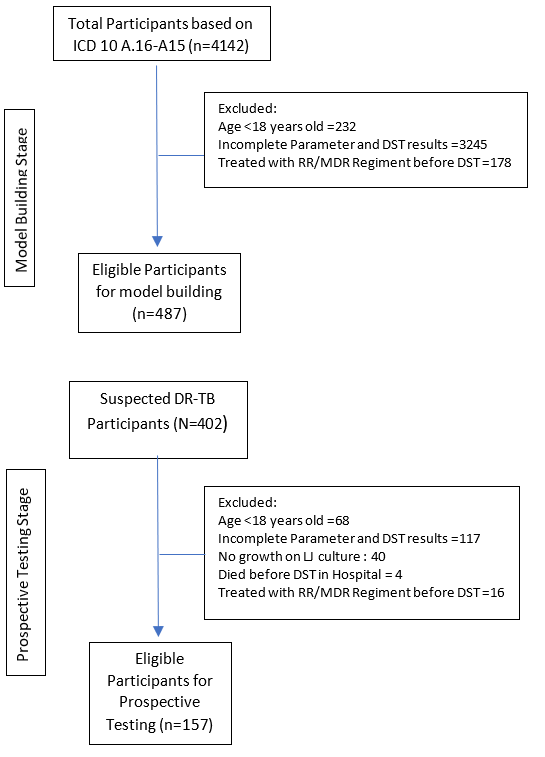

Supplement: S2 Fig — (DOCX) [file pone.0249243.s002.docx]

S4 Fig. Sensitivity Specificity of selected model (Artificial Neural Network Full Model 2-2)
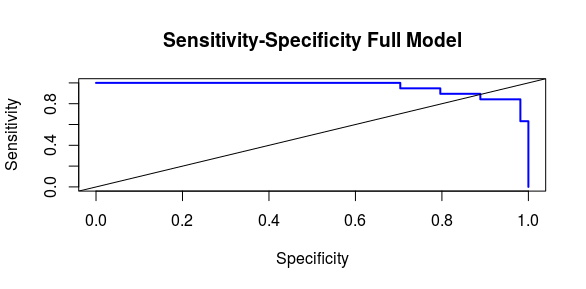

Supplement: S4 Fig — (DOCX) [file pone.0249243.s004.docx]

S6 Fig. Precision Recall of selected model (Artificial Neural Network Full Model 2-2).


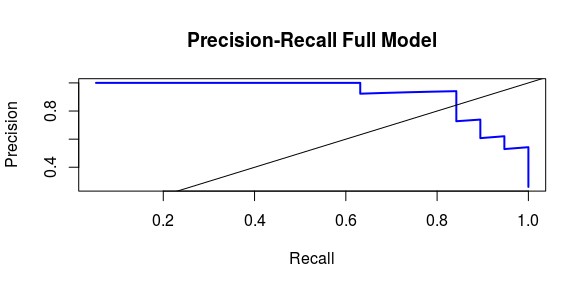

Supplement: S6 Fig — (DOCX) [file pone.0249243.s006.docx]

S7 Fig. CUHAS-ROBUST interface.


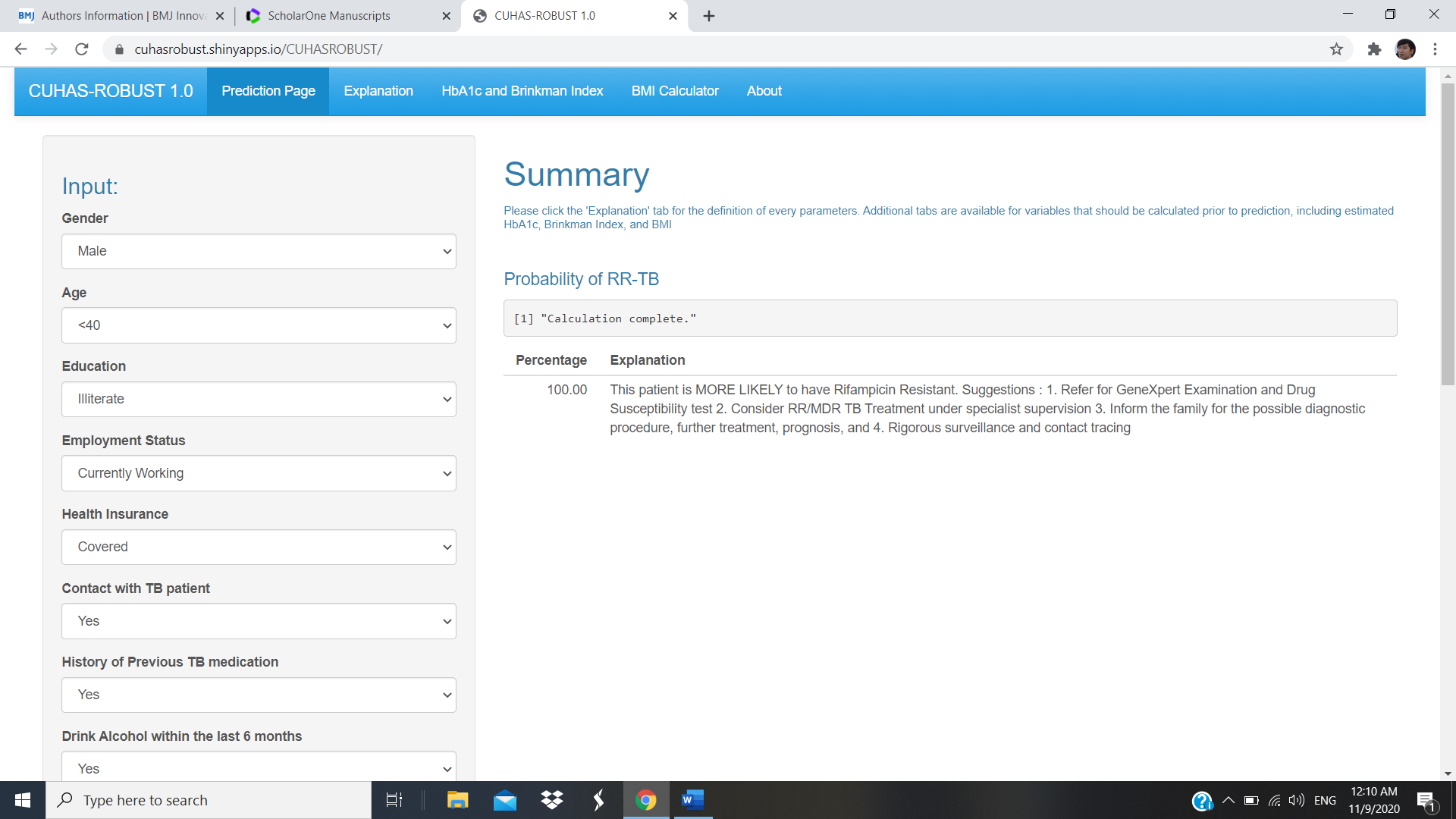

Supplement: S7 Fig — (DOCX) [file pone.0249243.s007.docx]

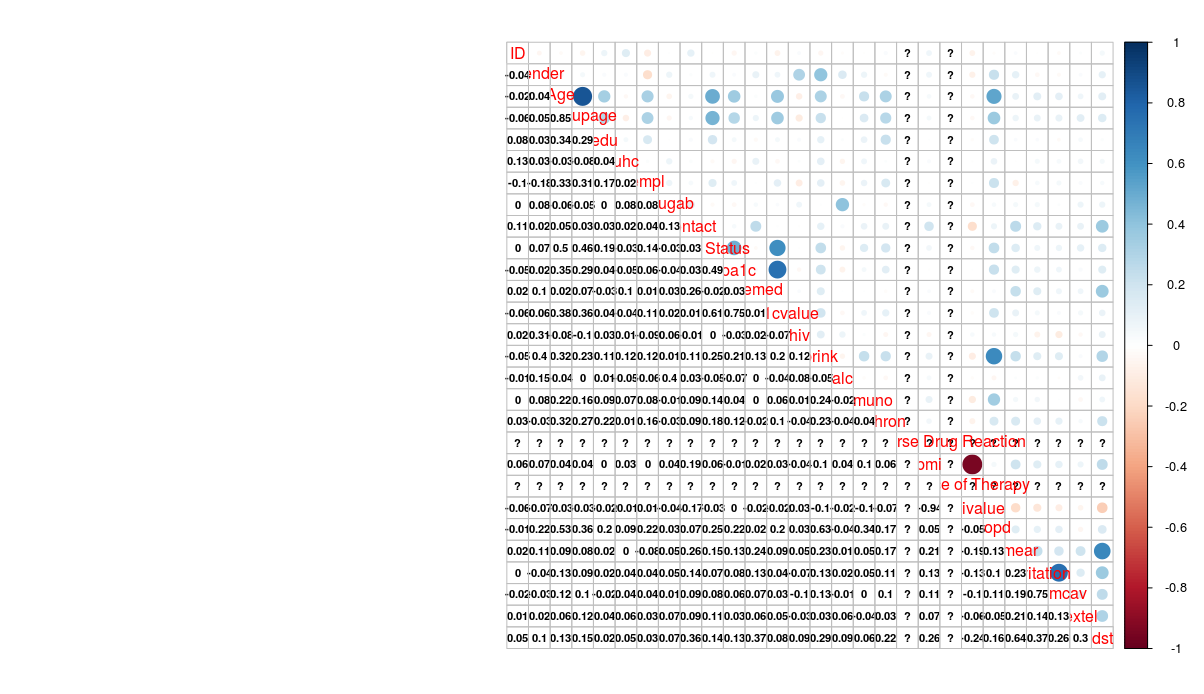

Supplement: S1 File — (ZIP) [file pone.0249243.s017.zip › S1_File/corrplot.png]

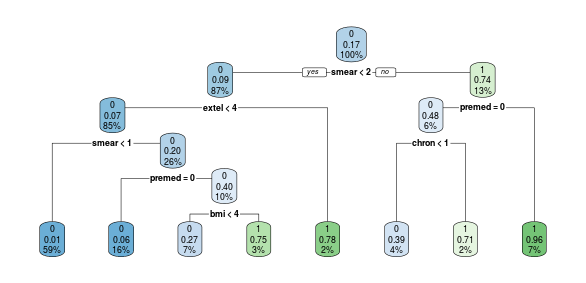

Supplement: S1 File — (ZIP) [file pone.0249243.s017.zip › S1_File/Decision Tree Bivariate Model Plot.png]

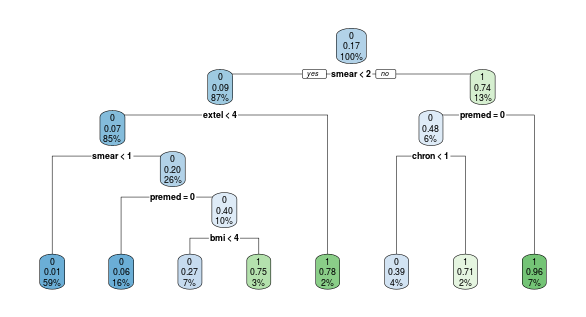

Supplement: S1 File — (ZIP) [file pone.0249243.s017.zip › S1_File/Decision Tree Full Model Plot.png]

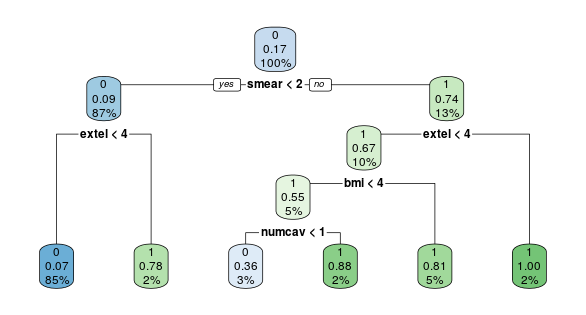

Supplement: S1 File — (ZIP) [file pone.0249243.s017.zip › S1_File/Decision Tree Short Model Plot.png]

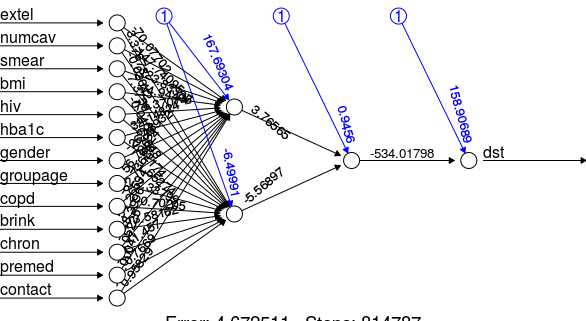

Supplement: S1 File — (ZIP) [file pone.0249243.s017.zip › S1_File/Plot Neural Network Bivariate Model 2-1.png]

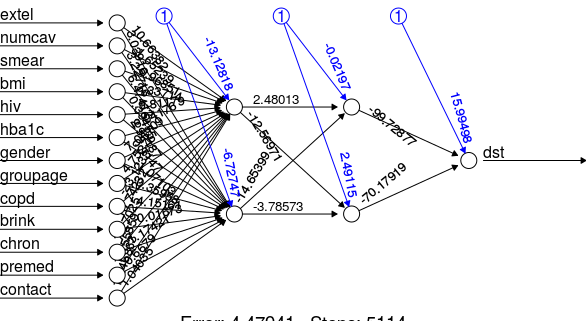

Supplement: S1 File — (ZIP) [file pone.0249243.s017.zip › S1_File/Plot Neural Network Bivariate Model 2-2.png]

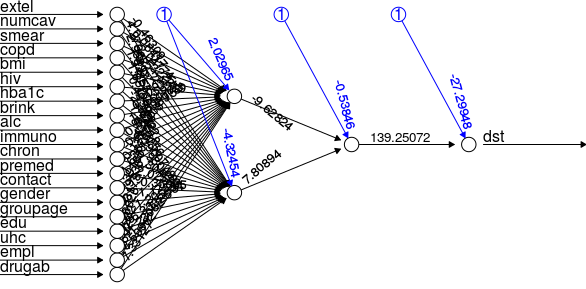

Supplement: S1 File — (ZIP) [file pone.0249243.s017.zip › S1_File/Plot Neural Network Full Model 2-1.png]

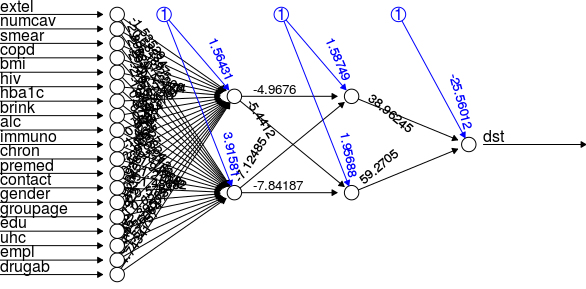

Supplement: S1 File — (ZIP) [file pone.0249243.s017.zip › S1_File/Plot Neural Network Full Model 2-2.png]

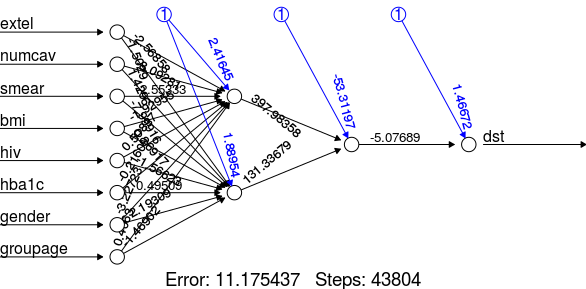

Supplement: S1 File — (ZIP) [file pone.0249243.s017.zip › S1_File/Plot Neural Network Short Model 2-1.png]

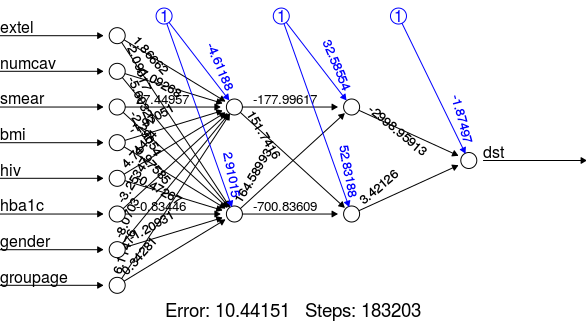

Supplement: S1 File — (ZIP) [file pone.0249243.s017.zip › S1_File/Plot Neural Network Short Model Model 2-2.png]

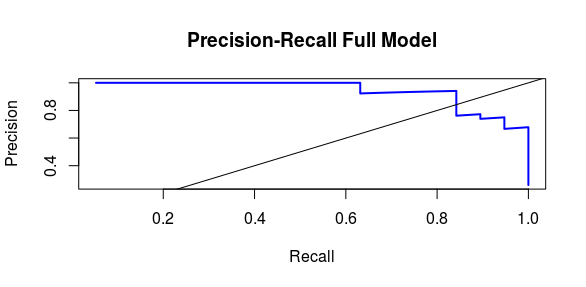

Supplement: S1 File — (ZIP) [file pone.0249243.s017.zip › S1_File/Prec-Recall Bivariate Model 2-1.png]

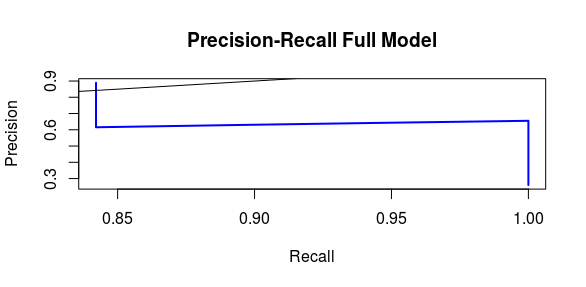

Supplement: S1 File — (ZIP) [file pone.0249243.s017.zip › S1_File/Prec-Recall Bivariate Model 2-2.png]

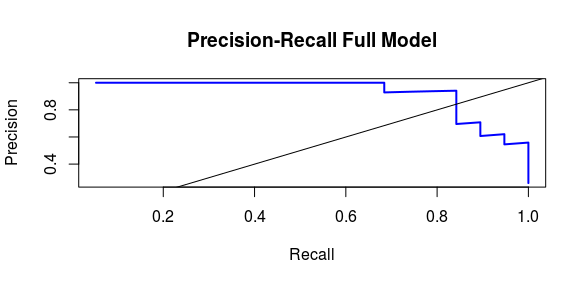

Supplement: S1 File — (ZIP) [file pone.0249243.s017.zip › S1_File/Prec-Recall Bivariate Model Logistic Regression.png]

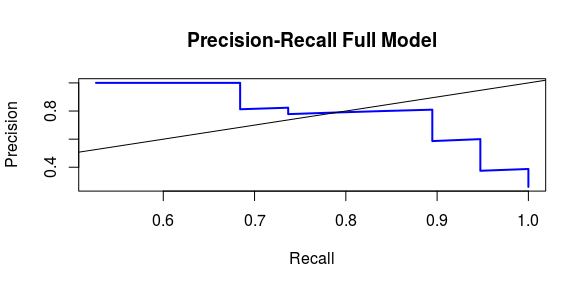

Supplement: S1 File — (ZIP) [file pone.0249243.s017.zip › S1_File/Prec-Recall Full Model 2-1.png]

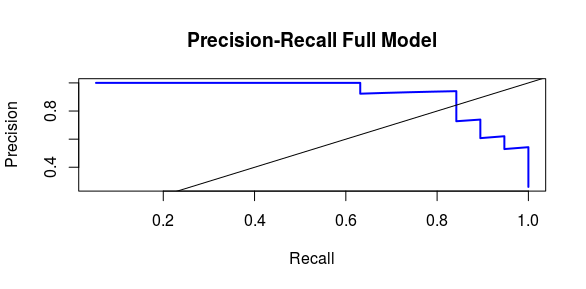

Supplement: S1 File — (ZIP) [file pone.0249243.s017.zip › S1_File/Prec-Recall Full Model 2-2.png]

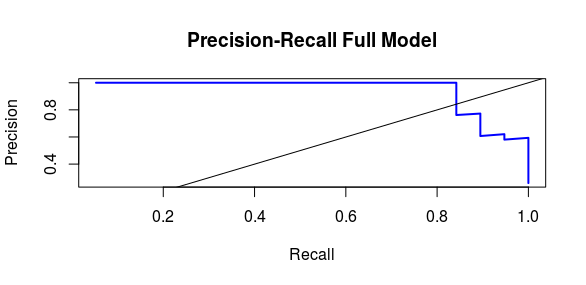

Supplement: S1 File — (ZIP) [file pone.0249243.s017.zip › S1_File/Prec-Recall Full Model Logistic Regression.png]

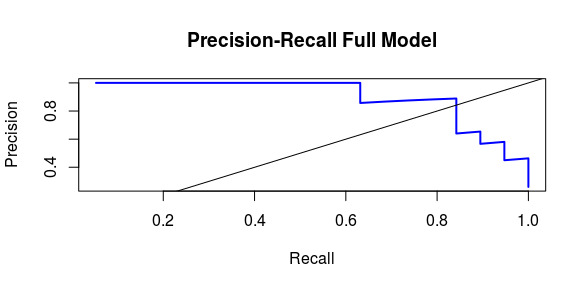

Supplement: S1 File — (ZIP) [file pone.0249243.s017.zip › S1_File/Prec-Recall Short Model 2-1.png]

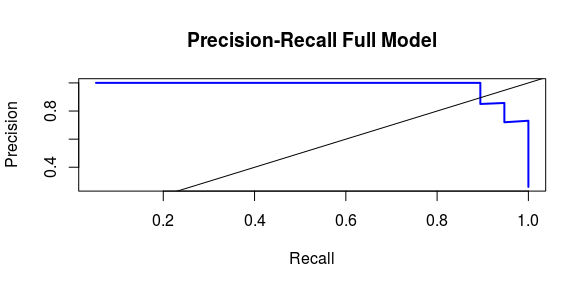

Supplement: S1 File — (ZIP) [file pone.0249243.s017.zip › S1_File/Prec-Recall Short Model 2-2.png]

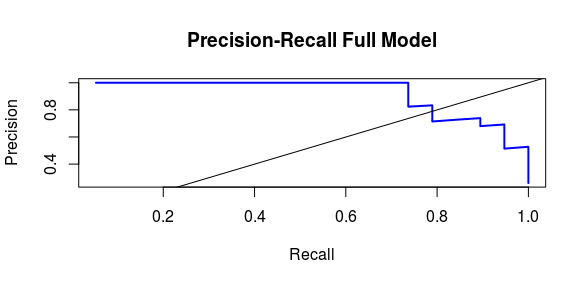

Supplement: S1 File — (ZIP) [file pone.0249243.s017.zip › S1_File/Prec-Recall Short Model Logistic Regression.png]

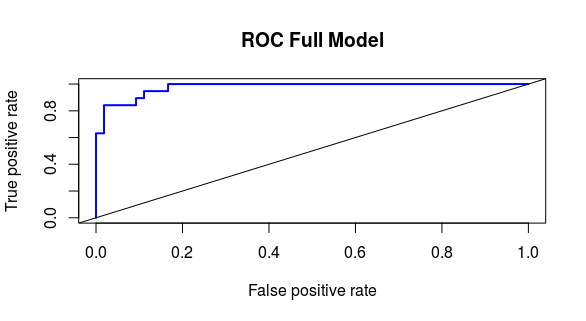

Supplement: S1 File — (ZIP) [file pone.0249243.s017.zip › S1_File/ROC Bivariate Model 2-1.png]

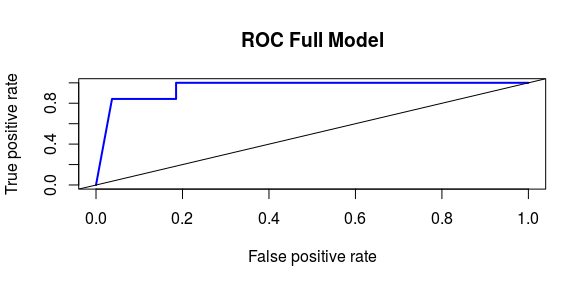

Supplement: S1 File — (ZIP) [file pone.0249243.s017.zip › S1_File/ROC Bivariate Model 2-2.png]

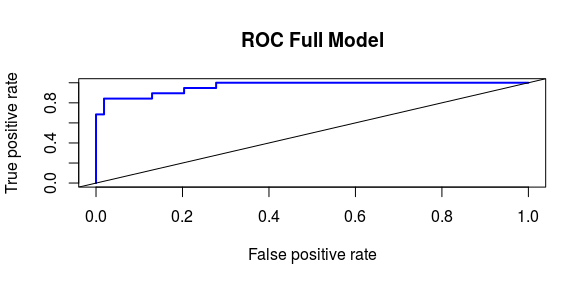

Supplement: S1 File — (ZIP) [file pone.0249243.s017.zip › S1_File/ROC Bivariate Model Logistic Regression.png]

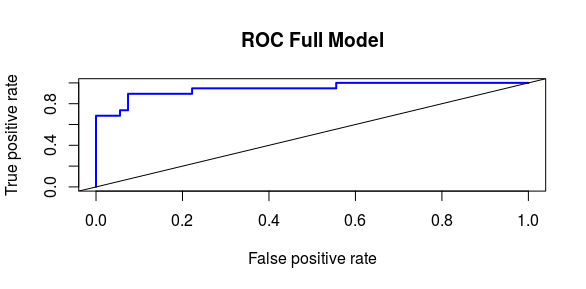

Supplement: S1 File — (ZIP) [file pone.0249243.s017.zip › S1_File/ROC Full Model 2-1.png]

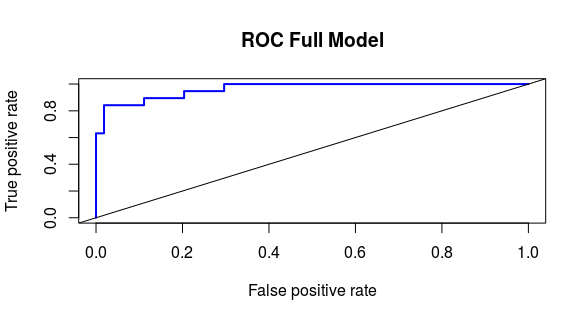

Supplement: S1 File — (ZIP) [file pone.0249243.s017.zip › S1_File/ROC Full Model 2-2.png]

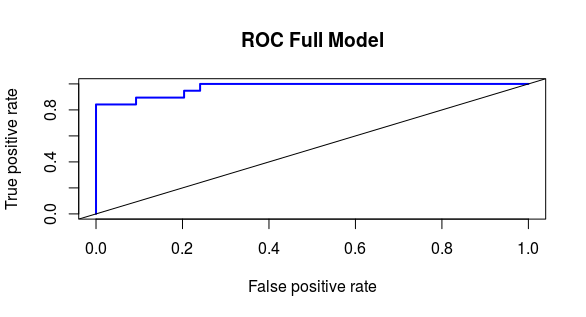

Supplement: S1 File — (ZIP) [file pone.0249243.s017.zip › S1_File/ROC Full Model Logistic Regression.png]

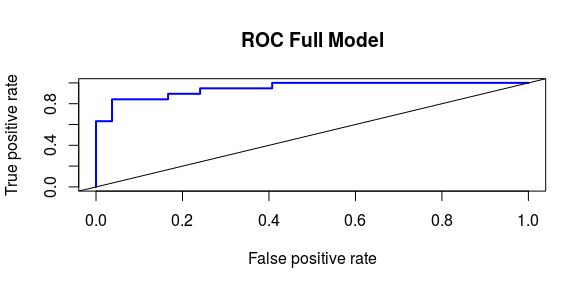

Supplement: S1 File — (ZIP) [file pone.0249243.s017.zip › S1_File/ROC Short Model 2-1.png]

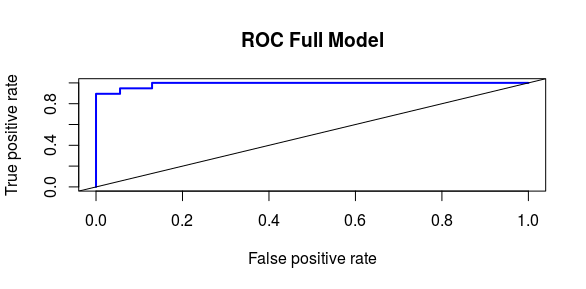

Supplement: S1 File — (ZIP) [file pone.0249243.s017.zip › S1_File/ROC Short Model 2-2.png]

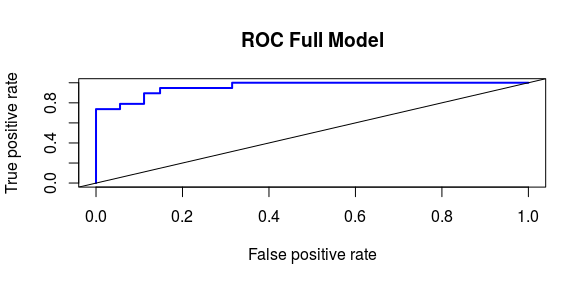

Supplement: S1 File — (ZIP) [file pone.0249243.s017.zip › S1_File/ROC Short Model Logistic Regression.png]

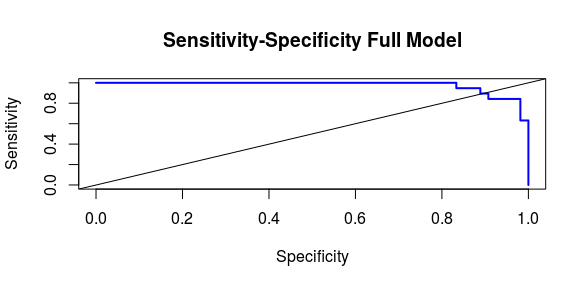

Supplement: S1 File — (ZIP) [file pone.0249243.s017.zip › S1_File/Sens-Spec Bivariate Model 2-1.png]

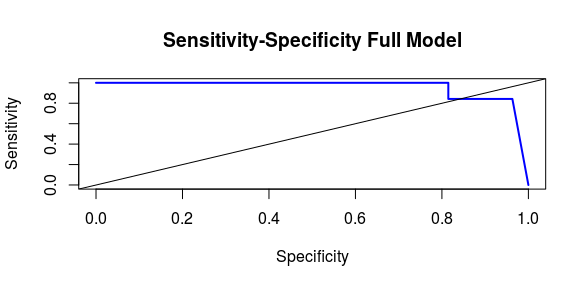

Supplement: S1 File — (ZIP) [file pone.0249243.s017.zip › S1_File/Sens-Spec Bivariate Model 2-2.png]

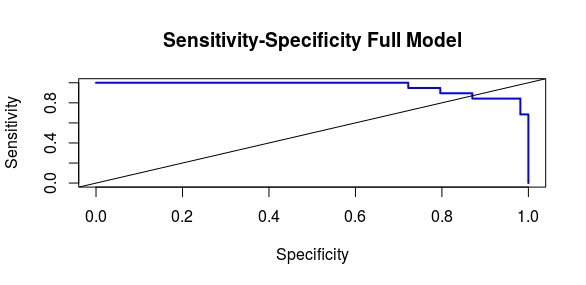

Supplement: S1 File — (ZIP) [file pone.0249243.s017.zip › S1_File/Sens-Spec Bivariate Model Logistic Regression.png]

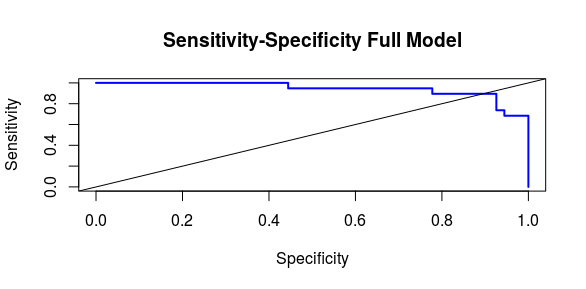

Supplement: S1 File — (ZIP) [file pone.0249243.s017.zip › S1_File/Sens-Spec Full Model 2-1.png]

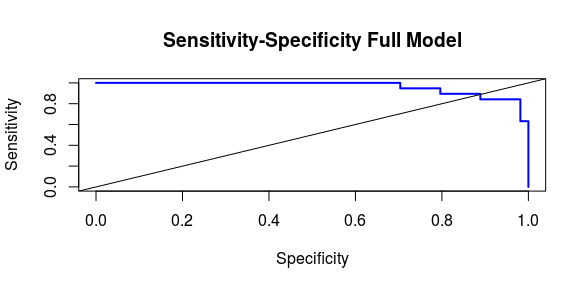

Supplement: S1 File — (ZIP) [file pone.0249243.s017.zip › S1_File/Sens-Spec Full Model 2-2.png]

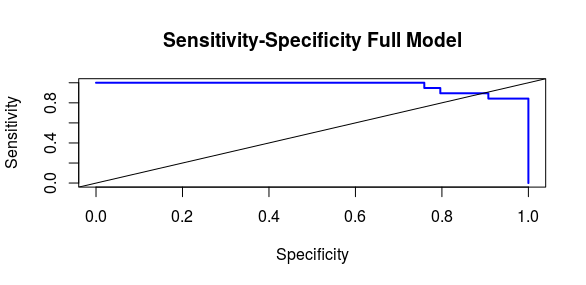

Supplement: S1 File — (ZIP) [file pone.0249243.s017.zip › S1_File/Sens-Spec Full Model Logistic Regression.png]

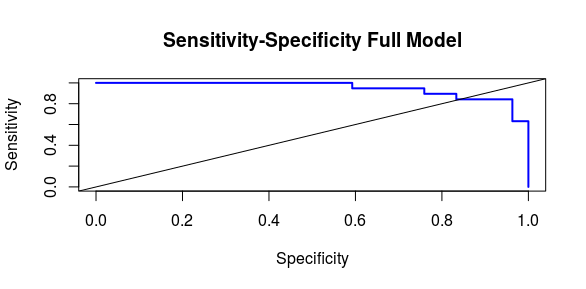

Supplement: S1 File — (ZIP) [file pone.0249243.s017.zip › S1_File/Sens-Spec Short Model 2-1.png]

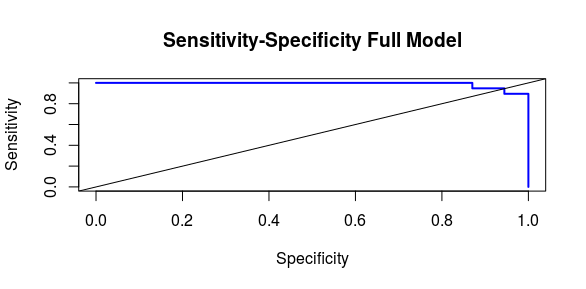

Supplement: S1 File — (ZIP) [file pone.0249243.s017.zip › S1_File/Sens-Spec Short Model 2-2.png]

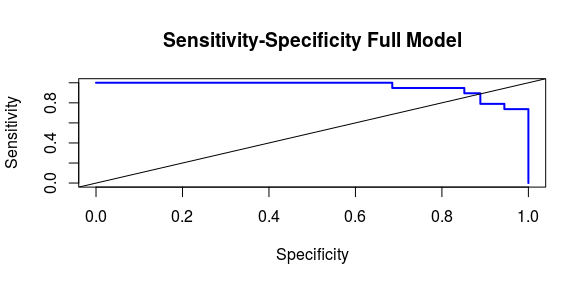

Supplement: S1 File — (ZIP) [file pone.0249243.s017.zip › S1_File/Sens-Spec Short Model Logistic Regression.png]

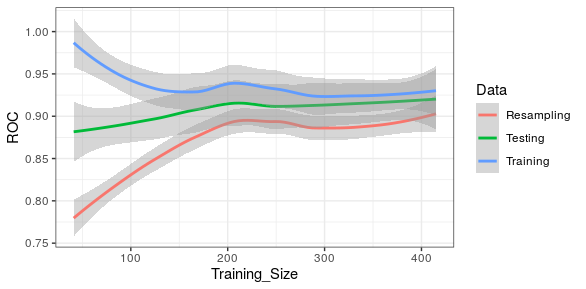

Supplement: S1 File — (ZIP) [file pone.0249243.s017.zip › S1_File/Training Size.png]

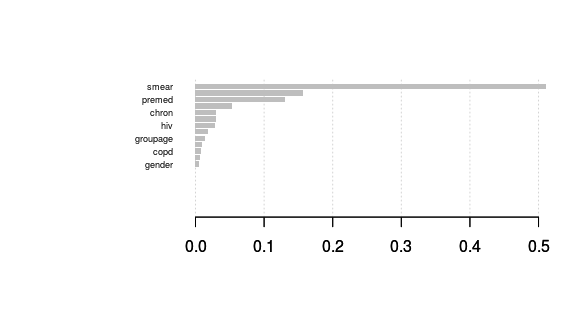

Supplement: S1 File — (ZIP) [file pone.0249243.s017.zip › S1_File/XGB importance Bivariate Model.png]

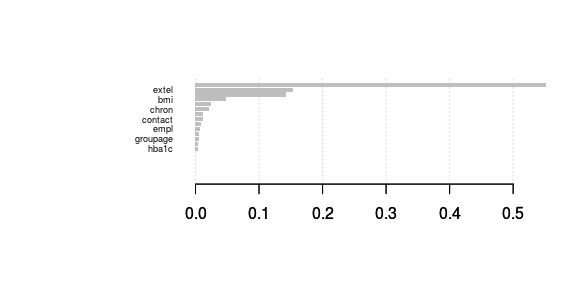

Supplement: S1 File — (ZIP) [file pone.0249243.s017.zip › S1_File/XGB importance Full Model.png]

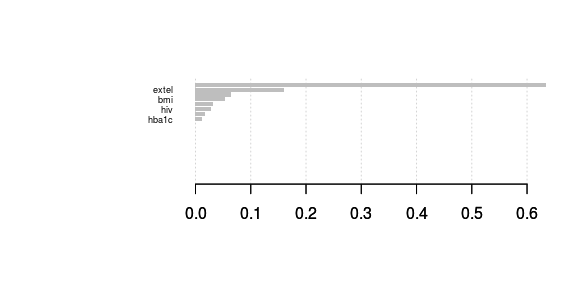

Supplement: S1 File — (ZIP) [file pone.0249243.s017.zip › S1_File/XGB importance Short Model.png]
