# Supplementary material for: Development and performance of CUHAS-ROBUST application for pulmonary rifampicin-resistance tuberculosis screening in Indonesia
Source: PLoS One. 2021 Mar 25;16(3):e0249243. doi: 10.1371/journal.pone.0249243 (PMC7993842; doi:10.1371/journal.pone.0249243)

S3 Fig. The Artificial Neural Network Structure of Full Model with two hidden layers and two nodes in each layer. The blue lines indicate the bias of each node.


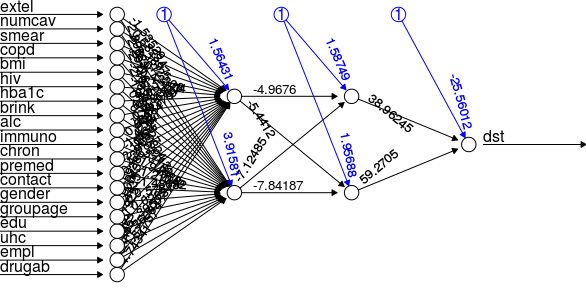

Supplement: S3 Fig — The blue lines indicate the bias of each node. (DOCX) [file pone.0249243.s003.docx]
