# Supplementary material for: Development and performance of CUHAS-ROBUST application for pulmonary rifampicin-resistance tuberculosis screening in Indonesia
Source: PLoS One. 2021 Mar 25;16(3):e0249243. doi: 10.1371/journal.pone.0249243 (PMC7993842; doi:10.1371/journal.pone.0249243)

S5 Fig. Receiver Operating Characteristic Curve of selected model (Artificial Neural Network Full Model 2-2)


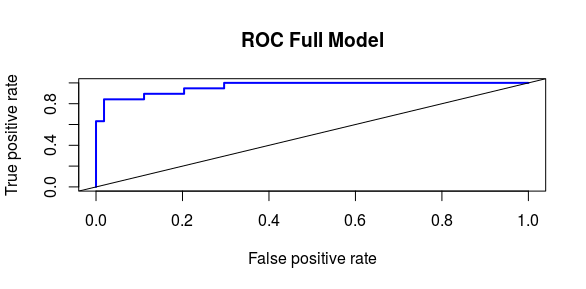

Supplement: S5 Fig — (DOCX) [file pone.0249243.s005.docx]
