# Supplementary material for: Development and performance of CUHAS-ROBUST application for pulmonary rifampicin-resistance tuberculosis screening in Indonesia
Source: PLoS One. 2021 Mar 25;16(3):e0249243. doi: 10.1371/journal.pone.0249243 (PMC7993842; doi:10.1371/journal.pone.0249243)
Supplement: S1 Table — (DOCX) [file pone.0249243.s008.docx]

| **S1 Table. Participant’s characteristic in model building data (n=487).** | | | | |
| --- | --- | --- | --- | --- |
| **Variable** | **Subset** | **RR + MDR (n=89)** | **Non-RR (n=398)** | **p value** |
| Gender | Male | 41 | 132 | 0.021 |
|  | Female | 48 | 266 |  |
| Age (year) | <40 | 30 | 210 | 0.001 |
|  | 40 and above | 59 | 188 |  |
|  | Mean ± SD | 44.06 ± 11.57 | 39.59 ± 13.84 |  |
| Education | Illiterate | 4 | 13 | 0.662^&^ |
|  | Primary Education | 19 | 95 |  |
|  | Secondary Education | 64 | 271 |  |
|  | College degree and above | 2 | 19 |  |
| Universal Health Coverage | Covered | 70 | 331 | 0.313 |
|  | Uncovered | 19 | 67 |  |
| Current Employment Status | Employed | 34 | 168 | 0.488 |
|  | unemployed | 55 | 230 |  |
| History of Drug Abuse | Never | 87 | 396 | 0.154^&^ |
|  | Yes | 2 | 2 |  |
| Contact with positive DR-TB case | Never | 61 | 381 | <0.001 |
|  | Yes | 28 | 17 |  |
| DM status | No | 40 | 252 | 0.001 |
|  | Yes | 49 | 146 | 0.004# |
|  | Mean ± SD of HbA1c | 7.33 ± 1.86 | 6.91 ± 1.95 |  |
| History of Previous TB treatment | Never | 27 | 299 | <0.001 |
|  | Yes | 62 | 99 |  |
| HIV status | Reactive | 26 | 77 | 0.039 |
|  | Non-Reactive | 63 | 321 |  |
| Brinkmann Index | Never Smoke | 53 | 340 | <0.001 |
|  | 1-600 | 27 | 55 |  |
|  | >600 | 9 | 3 |  |
| Drink alcohol within one year | Never | 86 | 395 | 0.078^&^ |
|  | yes | 3 | 3 |  |
| Immunosuppressants use > 6 weeks | Never | 85 | 390 | 0.245^&^ |
|  | Yes | 4 | 8 |  |
| Number of Chronic Disease | Median ± IQR | 0 ± 0 | 0 ± 0 | <0.001^ |
|  | Min-Max | 0-2 | 0-2 |  |
| Body Mass Index (kg/m2) | <18.5 | 49 | 74 | <0.001 |
|  | 18-5-<23 | 28 | 194 |  |
|  | 23-25 | 6 | 71 |  |
|  | >25 | 6 | 59 |  |
| Adherence to Previous TB treatment | Yes | 30 | 84 | <0.001 |
|  | No | 32 | 15 |  |
| Diagnosed as COPD | Yes | 21 | 40 | <0.001 |
|  | No | 68 | 358 |  |
| Sputum Smear level | Negative or Scanty | 3 | 285 |  |
|  | 1+ | 37 | 99 |  |
|  | 2+ | 32 | 9 | <0.001 |
|  | 3+ | 17 | 5 |  |
| Presence of Cavitation | Yes | 55 | 77 |  |
|  | No | 34 | 321 | <0.001 |
|  | Median Number ± IQR | 0 ± 2 | 0 ± 0 | <0.001^$^ |
|  | Min-Max of Cavitation | 0-4 | 0-4 |  |
| Extension of Lesion | Median ± IQR | 3 ± 1 | 2 ± 2 | <0.001^ |
|  | Min-Max | 1-4 | 0-4 |  |
| ^Abbreviation: COPD (Chronic Obstructive Pulmonary Disease), DM (Diabetes Mellitus), DR TB (Drug-Resistant Tuberculosis). DST (Drug Susceptibility Test), HbA1c (Hemoglobin Glycated 1c) HIV (Human Immunodeficiency Virus), IQR (Interquartile Range), Max (Maximum), MDR (multidrug-resistant) Min (Minimum), SD (Standard Deviation). All tested with Chi-Square, except (& = Fisher Exact). # is a Mann Whitney U test for the difference between HbA1c value. $ is a Mann Whitney test for difference of cavitation number between the group, ^ tested with Mann Whitney. The baseline for prospective testing data provides as supplement table 1^ | | | | |
