# Supplementary material for: Development and performance of CUHAS-ROBUST application for pulmonary rifampicin-resistance tuberculosis screening in Indonesia
Source: PLoS One. 2021 Mar 25;16(3):e0249243. doi: 10.1371/journal.pone.0249243 (PMC7993842; doi:10.1371/journal.pone.0249243)
Supplement: S2 Table — (DOCX) [file pone.0249243.s009.docx]

| **S2 Table. Descriptive Statistic of Prospective Data (n=157).** | | | | |
| --- | --- | --- | --- | --- |
| **Variable** | **Subset** | **RR + MDR (n=44)** | **Non-RR (n=113)** | **p value** |
| Gender | Male | 12 | 62 | 0.040 |
|  | Female | 32 | 51 |  |
| Age (year) | <40 | 7 | 30 | 0.158 |
|  | 40 and above | 37 | 83 |  |
|  | Mean ± SD | 54.79 ± 13.51 | 51.60 ± 16.29 |  |
| Education | Illiterate | 7 | 12 | 0.293 |
|  | Primary Education | 12 | 20 |  |
|  | Secondary Education | 12 | 46 |  |
|  | College degree and above | 13 | 35 |  |
| Universal Health Coverage | Covered | 33 | 86 | 0.884 |
|  | Uncovered | 11 | 27 |  |
| Current Employment Status | Employed | 26 | 66 | 0.938 |
|  | unemployed | 18 | 47 |  |
| History of Drug Abuse | Never | 41 | 112 | 0.067^&^ |
|  | Yes | 3 | 1 |  |
| Contact with positive DR-TB case | Never | 39 | 109 | 0.118^&^ |
|  | Yes | 5 | 4 |  |
| HbA1c | <6.5 | 38 | 92 | 0.461 |
|  | >6.5 | 6 | 21 |  |
| History of Previous TB treatment | Never | 20 | 81 | 0.002 |
|  | Yes | 24 | 32 |  |
| HIV status | Reactive | 2 | 2 | 0.313^&^ |
|  | Non-Reactive | 42 | 111 |  |
| Brinkmann Index | Never Smoke | 19 | 92 | <0.001^&^ |
|  | 1-600 | 23 | 20 |  |
|  | >600 | 2 | 1 |  |
| Drink alcohol within one year | Never | 41 | 113 | 0.021^&^ |
|  | yes | 3 | 0 |  |
| Immunosuppressants use > 6 weeks | Never | 38 | 105 | 0.218^&^ |
|  | Yes | 6 | 8 |  |
| Number of Chronic Disease | 0 | 25 | 64 | 1.000^&^ |
|  | 1 | 19 | 47 |  |
|  | 2 | 0 | 2 |  |
| Body Mass Index (kg/m2) | <18.5 | 16 | 37 | 0.315^&^ |
|  | 18-5-<23 | 24 | 67 |  |
|  | 23-25 | 1 | 7 |  |
|  | >25 | 3 | 2 |  |
|  |  |  |  |  |
| Diagnosed as COPD | Yes | 4 | 4 | 0.221^&^ |
|  | No | 40 | 109 |  |
| Sputum Smear level | Negative or Scanty | 14 | 45 |  |
|  | 1+ | 16 | 62 |  |
|  | 2+ | 7 | 5 | <0.001^&^ |
|  | 3+ | 7 | 1 |  |
| Presence of Cavitation | Yes | 9 | 2 |  |
|  | No | 35 | 101 | <0.001^&^ |
| Extension of Lesion | 0  1  2  3  4  5  6 | 11  9  11  5  4  1  3 | 81  18  11  2  1  0  0 | <0.001^&^ |
| ^Abbreviation: COPD (Chronic Obstructive Pulmonary Disease), DM (Diabetes Mellitus), DR TB (Drug-Resistant Tuberculosis). DST (Drug Susceptibility Test), HbA1c (Hemoglobin Glycated 1c) HIV (Human Immunodeficiency Virus), IQR (Interquartile Range), Max (Maximum), MDR (multidrug-resistant) Min (Minimum), SD (Standard Deviation). All tested with Chi-Square, except (& = Fisher Exact)^ | | | | |
