# Supplementary material for: Development and performance of CUHAS-ROBUST application for pulmonary rifampicin-resistance tuberculosis screening in Indonesia
Source: PLoS One. 2021 Mar 25;16(3):e0249243. doi: 10.1371/journal.pone.0249243 (PMC7993842; doi:10.1371/journal.pone.0249243)
Supplement: S3 Table — (DOCX) [file pone.0249243.s010.docx]

**S3 Table. List of Variables.**

| **Number** | **Variable** | **p-Value** |
| --- | --- | --- |
| 1 | Gender is defined as sex at birth. | 0.021 |
| 2 | Groupage is defined as below 40 years old and/or above 40 years old at the time of assessment. | 0.001 |
| 3 | Education is defined as the latest level attained. Ranging from illiterate, primary school, secondary school and college degree. | 0.662* |
| 4 | Employment status is defined as having a job with specific working hours. Freelance can be stated as currently working if the job provides a stable income flow. | 0.488 |
| 5 | Health insurance is defined as the holder of active private or national health insurance. | 0.313 |
| 6 | Contact with a TB patient is defined as any history of close contact with a positive TB patient. | <0.001 |
| 7 | History of Previous Medication defined as any TB treatment taken in the past (except prophylaxis). | <0.001 |
| 8 | Drink alcohol refers to any episode of alcohol drinking within the last 6 months, irrespective of the number of standards drinking. | 0.078* |
| 9 | Any use of an immunosuppressive agent including steroid (oral, injection, topical, or inhalant preparation), and anti-cancer therapy, or any other immunosuppressive agent for more than 6 weeks should be treated yes. | 0.245* |
| 10 | Brinkmann index defined as the number of cigarettes smoked times the year of smoking | <0.001 |
| 11 | HIV status is defined as any positive results of HIV testing either from a rapid serology test or ELISA. | 0.039 |
| 12 | Chronic Obstructive Pulmonary Disease demonstrates a lower forced expiration volume in spirometry. The GOLD (Global Initiative for Chronic Obstructive Lung Disease) classification is implemented for diagnosis. The ICD 10 code for the disease is J.44 | <0.001 |
| 13 | Any history of drug abuse in the past | 0.154* |
| 14 | The number of chronic diseases in the patient such as cancer, hypertension, autoimmune (exclude DM, COPD, HIV). | <0.001# |
| 15 | The hbA1c value from an NGSP-certified laboratory which represents the average of blood glucose level over the last three months. | 0.004 |
| 16 | The smear is defined as the results of sputum smear before DST and treatment. It is based on the number of bacilli ranging from negative, scanty, 1+ to 3+ | <0.001# |
| 17 | Number of cavities defined as the observed number of cavitation either in CT-Scan or Chest Xray, irrespective of the size, diameter, and wall thickness | <0.001# |
| 18 | The radiology appearance of the lung is divided into three sections (upper, middle, lower) per lung with a total of 6 sections. Specific findings of pulmonary tuberculosis in this application are cavity, Gohn Focus, consolidation, atelectasis, fibrotic line, hilar lymphadenopathy, milliary appearance, or pleural effusion. Any signs of this in one section are considered as one section, two findings in two different sections, the extension of the lesion considered as two sections. Two findings in one section are considered as one section. Each person has a maximum of six sections | <0.001# |
| 19 | The body mass index defined as body weight in kilograms per square of height in meters  Under 18.5    = Underweight 18.5–22.99   = normal weight 23.0–24.99   = overweight >25              = obese | <0.001 |
| 20 | Drug Suscecptibility Test   1. A standard procedure for treating the sputum and DST process was applied. Two samples of the morning and random sputum were digested with N-acetyl-L-cysteine and sodium hydroxide and concentrated. A 0.2 ml of suspension was embedded in the LJ medium and observed for any growth in a weekly interval. The culture with positive growth was then tested for DST with rifampicin concentration 1μg/mL. The cutoff of this method for resistance is >1% 2. A proportion method is a protocol implemented over the last five years in these centers and compared to the critical concentration method, the latter method is prone to underdiagnosis due to different responses of minimum inhibitory concentration in comparison with standard strain. 3. Cases with no growth from multiple samples in the initial LJ culture will be excluded. as it indicates an error in laboratory procedure or the sample collection, or the patient was not infected with TB. |  |
| Footnote:  Full Model: All Variables  Short Model: 1,2,11,15,16,17,18,19  Bivariate Model: 1,2,6,7,10,11,12,14,15,16,17,18,19  The P Value Shows bivariate analysis of these variables with the Drug Susceptibility Result  All tested with Chi Square Except:  *Fisher Exact  # Mann Whitney U | | |
