# Supplementary material for: Development and performance of CUHAS-ROBUST application for pulmonary rifampicin-resistance tuberculosis screening in Indonesia
Source: PLoS One. 2021 Mar 25;16(3):e0249243. doi: 10.1371/journal.pone.0249243 (PMC7993842; doi:10.1371/journal.pone.0249243)
Supplement: S4 Table — (DOCX) [file pone.0249243.s011.docx]

**S4 Table. Mathematical Equation of the model**

| **Number** | **Variable** | **Equation** |
| --- | --- | --- |
| Eq. B.1 | Neuron label | j |
| Eq. B.2 | Received input from predecessor neuron (net input) | p_j_(t) |
| Eq. B.3 | Activation | a_j_(t) |
| Eq. B.4 | Threshold | θ _j_(t) |
| Eq. B.5 | Activation function | f |
| Eq. B.6 | Computing new activation at given time (t+1) from activation and the net input | a_j_(t+1)=f(a_j_(t), θ _j,_ p_j_(t)) |
| Eq. B.7 | Output | o_j_(t)=f_out_(a_j_(t)) |
| Eq. B.8 | Propagation (computes input to the neuron of j based on output) | p_j_(t)=$\sum_{i} Oi\left( t \right).Wij$  Where:  W = weight |
| Eq. B.9 | Adding bias | p_j_(t)=$\sum_{i} Oi\left( t \right).Wij$ + W0j  Where:  W0j = bias |
| Eq. B.10 | Normalization Min-Max | $x scaled= \frac{X-Xmin}{Xmax-Xmin}$  Where:  X = The value of data  X min = the minimum value of X  X max = the maximum value of X  X scaled = new normalized X value |
| Eq. B.11 | Activation Function Sigmoid/Logistic (f) | $f\left( x \right)= \frac{1}{1+e^{-(x)}}$  Where  X = the weighted value |
| Eq.B.12 | Cross Entropy for Binary | $Cross entropy=-(ylog(p)+(1-y)log(1-p))$  Where:  log = the natural log  y = binary indicator (0 or 1)  p = predicted probability |
