# Supplementary material for: Development and performance of CUHAS-ROBUST application for pulmonary rifampicin-resistance tuberculosis screening in Indonesia
Source: PLoS One. 2021 Mar 25;16(3):e0249243. doi: 10.1371/journal.pone.0249243 (PMC7993842; doi:10.1371/journal.pone.0249243)
Supplement: S5 Table — (DOCX) [file pone.0249243.s012.docx]

**S5 Table. Structure of the Artificial Neural Network Model (ANN) (R Script available upon request).**

| Model | Predictor | Hidden Layer 1 | Hidden Layer 2 | Maximum Steps | Repetition |
| --- | --- | --- | --- | --- | --- |
| ANN Full Model 2-1 | 19 | 2 Nodes | 1 Node | 100000 | 20 |
| ANN Full Model 2-2 | 19 | 2 Nodes | 2 Nodes | 100000 | 20 |
| ANN Short Model 2-1 | 8 | 2 Nodes | 1 Node | 1000000 | 10 |
| ANN Short Model 2-2 | 8 | 2 Nodes | 2 Nodes | 1000000 | 10 |
| ANN Bivariate Model 2-2 | 13 | 2 Nodes | 2 Nodes | 100000 | 20 |
| ANN Bivariate Model 2-1 | 13 | 2 Nodes | 1 Nodes | 1000000 | 10 |
| Footnote: set seed (123); Normalization using min-max; Train/Test = 85%:15% using train-test split; training using neuralnet (resilient backpropagation); default threshold 0.01; activation function: Logistic; Loss: Cross Entropy | | | | | |
