# Supplementary material for: Development and performance of CUHAS-ROBUST application for pulmonary rifampicin-resistance tuberculosis screening in Indonesia
Source: PLoS One. 2021 Mar 25;16(3):e0249243. doi: 10.1371/journal.pone.0249243 (PMC7993842; doi:10.1371/journal.pone.0249243)
Supplement: S6 Table — (DOCX) [file pone.0249243.s013.docx]

**S6 Table. Structure of other Classifiers (R Script available upon request)**

| Model | Predictor | General Protocol |
| --- | --- | --- |
| Logistic Regression 1 | 19 | Function: glm.fit, Family binomial, Calculating LogLoss using Cross Entropy |
| Logistic Regression 2 | 8 |  |
| Logistic Regression 3 | 13 |  |
| Extreme Gradient Boost 1 | 19 | Function= xgboost. Using one hot encoding for creating dummy variable. Parameters consists of gbtree booster with eta = 0.3, no gamma regularization with maximum depth of tree = 6, number of rounds = 100 with 5 cross validation and early stopping rounds function. Selection was based on least error. |
| Extreme Gradient Boost 1 | 8 |  |
| Extreme Gradient Boost 1 | 13 |  |
| Decision Tree 1 | 19 | Function: rpart, method=class. Accuracy tuning with rpart.control was prepared with minimum observation exist in the node=4, with complexity parameter=0 |
| Decision Tree 2 | 8 |  |
| Decision Tree 3 | 13 |  |
| Random Forest 1 | 19 | Function= randomforest. Tuning parameter= mtry. Crossvalidation=5 |
| Random Forest 2 | 8 |  |
| Random Forest 3 | 13 |  |
| Common protocol : seed before data splitting 123. 85%:15% train:test splitting | | |
