# Supplementary material for: Development and performance of CUHAS-ROBUST application for pulmonary rifampicin-resistance tuberculosis screening in Indonesia
Source: PLoS One. 2021 Mar 25;16(3):e0249243. doi: 10.1371/journal.pone.0249243 (PMC7993842; doi:10.1371/journal.pone.0249243)
Supplement: S7 Table — (DOCX) [file pone.0249243.s014.docx]

**S7 Table. Performance of Artificial Neural Network model with 15% training data (N=73)**

| Model | TN | TP | FP | FN | %Acc  (95% CI) | % Sens (95% CI) | % Spec  (95% CI) | LogLoss | AUC |
| --- | --- | --- | --- | --- | --- | --- | --- | --- | --- |
| 2.2 Full | 51 | 16 | 3 | 3 | 92 (83-97) | 84 (60-97) | 94 (85-99) | 1.53 | 0.96 |
| 2.1 Full | 51 | 13 | 3 | 6 | 88 (78-94) | 68 (43-87) | 94 (85-99) | 1.56 | 0.94 |
| 2.1 Short | 52 | 13 | 2 | 6 | 89 (79-95) | 68 (43-87) | 96 (87-99) | 0.91 | 0.95 |
| 2.2 Short | 54 | 16 | 0 | 3 | 96 (88-99) | 84 (60-97) | 100 (93-100) | 0.18 | 0.99 |
| Bivariate 2-2 | 50 | 16 | 4 | 3 | 90(81-96) | 84 (60-97) | 93(82-98) | 2.81 | 0.96 |
| Bivariate 2-1 | 51 | 16 | 3 | 3 | 92(83-97) | 84 (60-97) | 94 (85-99) | 1.25 | 0.98 |
| Abbreviation: Acc = Accuracy; AUC = Area Under Curve; CI = Confidence Interval; FN = False Negative; FP = False Positive; TN = True Negative; TP = True Positive | | | | | | | | | |
