# Supplementary material for: Development and performance of CUHAS-ROBUST application for pulmonary rifampicin-resistance tuberculosis screening in Indonesia
Source: PLoS One. 2021 Mar 25;16(3):e0249243. doi: 10.1371/journal.pone.0249243 (PMC7993842; doi:10.1371/journal.pone.0249243)
Supplement: S8 Table — (DOCX) [file pone.0249243.s015.docx]

**S8 Table. Performance of Other models with 15% training data (N=73).**

| Model | TN | TP | FP | FN | %Acc  (95% CI) | % Sens (95% CI) | % Spec  (95% CI) |
| --- | --- | --- | --- | --- | --- | --- | --- |
| LR full | 53 | 16 | 1 | 3 | 95(87-98) | 84 (60-97) | 98(90-99) |
| LR Bivariate | 52 | 16 | 2 | 3 | 93(85-98) | 84 (60-97) | 96 (87-99) |
| LR Short | 54 | 14 | 0 | 5 | 93(85-98) | 74(45-91) | 100 (93-100) |
| DT Full | 54 | 13 | 0 | 6 | 92(83-97) | 68 (43-87) | 100 (93-100) |
| DT Bivariate | 54 | 13 | 0 | 6 | 92(83-97) | 68 (43-87) | 100 (93-100) |
| DT Short | 54 | 10 | 0 | 9 | 88(78-94) | 53(29-76) | 100 (93-100) |
| RF Full | 54 | 15 | 0 | 4 | 95(87-98) | 79(54-94) | 100 (93-100) |
| RF Bivariate | 53 | 15 | 1 | 4 | 93(85-98) | 79(54-94) | 98(90-99) |
| RF Short | 54 | 12 | 0 | 7 | 90(81-96) | 63(38-84) | 100 (93-100) |
| XGB Full | 54 | 14 | 0 | 5 | 93(85-98) | 74(49-91) | 100 (93-100) |
| XGB Bivariate | 54 | 15 | 0 | 4 | 95(87-98) | 79(54-94) | 100 (93-100) |
| XGB Short | 54 | 14 | 0 | 5 | 93(85-98) | 74(49-91) | 100 (93-100) |
| Abbreviation: Acc = Accuracy; AUC = Area Under Curve; CI = Confidence Interval; DT = Decision Tree; FN = False Negative; FP = False Positive; LR = Logistic Regression; RF = Random Forest; TN = True Negative; TP = True Positive; XGB = Extreme Gradient Boost | | | | | | | |
