# Supplementary material for: Development and performance of CUHAS-ROBUST application for pulmonary rifampicin-resistance tuberculosis screening in Indonesia
Source: PLoS One. 2021 Mar 25;16(3):e0249243. doi: 10.1371/journal.pone.0249243 (PMC7993842; doi:10.1371/journal.pone.0249243)
Supplement: S9 Table — (DOCX) [file pone.0249243.s016.docx]

**S9 Table. Performance of all models from prospective data (N=157).**

| MODEL | TN | TP | FP | FN | %Accuracy  (95% CI) | % Sensitivity (95% CI) | % Specificity  (95% CI) |
| --- | --- | --- | --- | --- | --- | --- | --- |
| ANN 2.2 Full | 104 | 37 | 9 | 7 | **90(84-94)** | 84(70-93) | 92(85-96) |
| ANN 2.1 Full | 62 | 41 | 51 | 3 | 66(58-73) | 93(81-98) | 55(45-64) |
| ANN 2.1 Short | 66 | 33 | 47 | 11 | 63(55-71) | 75(60-87) | 58(49-68) |
| ANN 2.2 Short | 0 | 44 | 113 | 0 | 28(21-36) | **100(92-100)** | 0(0-3) |
| Bivariate 2-2 | 58 | 37 | 55 | 7 | 61(52-68) | 84(70-93) | 51(42-61) |
| Bivariate 2-1 | 27 | 43 | 86 | 1 | 45(37-53) | 98(88-99) | 24(16-34) |
| LR full | 112 | 17 | 1 | 27 | 82(75-88) | 39(24-54) | **99(95-99)** |
| LR Bivariate | 112 | 18 | 1 | 26 | 83(76-88) | 41(26-57) | **99(95-99)** |
| LR Short | 112 | 14 | 1 | 30 | 80(73-86) | 32(19-48) | **99(95-99)** |
| DT Full | 106 | 22 | 7 | 22 | 82(75-87) | 50(35-65) | 94(88-97) |
| DT Bivariate | 106 | 22 | 7 | 22 | 82(75-87) | 50(35-65) | 94(88-97) |
| DT Short | 110 | 16 | 3 | 28 | 80(73-86) | 36(22-52) | 97(92-99) |
| RF Full | 107 | 13 | 6 | 31 | 76(69-83) | 30(17-45) | 95(89-98) |
| RF Bivariate | 107 | 14 | 6 | 30 | 77(70-83) | 32(19-48) | 95(89-98) |
| RF Short | 110 | 10 | 3 | 34 | 76(69-83) | 23(11-38) | 97(92-99) |
| XGB Full | 108 | 8 | 5 | 36 | 74(66-81) | 18(8-33) | 96(90-99) |
| XGB Bivariate | 107 | 9 | 6 | 35 | 74(66-81) | 20(10-35) | 95(89-98) |
| XGB Short | 107 | 9 | 6 | 35 | 74(66-81) | 20(10-35) | 95(89-98) |
| Abbreviation:  Acc = Accuracy  ANN = Artificial Neural Network  CI = Confidence Interval  DT = Decision Tree  FN = False Negative  FP = False Positive  LR = Logistic Regression  RF = Random Forest  TN = True Negative  TP = True Positive  XGB = Extreme Gradient Boost | | | | | | | |
